# Quercetin and Its Nano‐Based Formulations Against Skin Cancer: A Narrative Review

**DOI:** 10.1002/hsr2.71920

**Published:** 2026-02-28

**Authors:** Mahtab Khanyabzadeh, Alireza Emamifar, Nikoo Emtiazi, Amir Nazari, Masoumeh Shekarriz, Amin Karami, Fateme Hashem Beik Mahallati, Rahineh Nomani Lafmejani, Atieh Dariush, Seyed Mohammad Mahdi Rohani, Elaheh Mohseni Vadeghani, Negin Khoshnood, Hamid Reza Ojaghi, Fatemeh Rezaei‐Tazangi, Reza Arefnezhad

**Affiliations:** ^1^ Dezful University of Medical Sciences Ahvaz Iran; ^2^ Department of Biology Azad University, Rasht Branch Rasht Gilan Iran; ^3^ Department of Pathology Firoozgar Hospital, Iran University of Medical Sciences Tehran Iran; ^4^ Shahid Beheshti University of Medical Sciences Tehran Iran; ^5^ Department of Biotechnology School of Biology, College of Science, University of Tehran Tehran Iran; ^6^ Department of Orthopedic Surgery Clinical Research Development Unit of Shohada‐e Tajrish Hospital, Shahid Beheshti University of Medical Sciences Tehran Iran; ^7^ Wageningen University and Research Wageningen Gelderland Netherlands; ^8^ Yazd Shahid Sadoughi University of Medical Sciences Yazd Iran; ^9^ Tehran University of Medical Sciences Tehran Iran; ^10^ Faculty of Science and Convergence, Science and Research Unit, Islamic Azad University Tehran Iran; ^11^ Department of Dermatology Tabriz University of Medical Sciences Tabriz Iran; ^12^ Department of Anatomy School of Medicine, Fasa University of Medical Sciences Fasa Iran; ^13^ Student Research Committee Shiraz University of Medical Sciences Shiraz Iran

**Keywords:** basal cell carcinoma, melanoma, nanotechnology, Quercetin, skin cancer, squamous cell carcinoma

## Abstract

**Background:**

Skin cancer is one of the most prevalent malignancies worldwide, characterized by the abnormal growth of skin cells and significant clinical challenges. Conventional treatments—including surgery, chemotherapy, and radiotherapy—often suffer from limitations such as adverse side effects, tumor resistance, and inadequate efficacy. In this context, natural compounds have garnered attention as alternative therapeutic agents. Quercetin, a flavonoid widely distributed in fruits and vegetables, is recognized for its anti‐cancer, anti‐inflammatory, and antioxidant properties. However, its clinical application is hindered by poor solubility, low bioavailability, and limited skin permeation. Recent advances in nanotechnology have led to the development of nano‐based formulations that can enhance the pharmacological performance of quercetin, offering promising avenues for skin cancer management.

**Objective:**

This review aims to provide a comprehensive analysis of skin cancer pathogenesis and to evaluate the mechanistic insights and therapeutic potential of quercetin—alone and in nano‐formulated systems—in preclinical models. The novelty of this review lies in its integrated approach, combining an overview of natural compound therapy with the latest cutting‐edge nanotechnology strategies to overcome current treatment challenges.

**Methods:**

This literature review was performed by searching related words, including “Skin cancer,” “Melanoma,” “Basal cell carcinoma,” “Squamous cell carcinoma,” “Quercetin,” “Nanotechnology,” in different databases like Google Scholar, Scopus, PubMed, Web of Science, and Scientific Information Databases until 2025.

**Results:**

Preclinical studies indicate that quercetin modulates multiple cellular and molecular pathways—such as those regulating apoptosis, cell cycle progression, mitochondrial function, and DNA repair—to inhibit proliferation, migration, and invasion of skin cancer cells. Nano‐based delivery systems (e.g., titanium dioxide nanoparticles, nanogels, and lipid carrier gels) have been shown to further enhance these therapeutic effects by improving quercetin's stability, skin permeability, and bioavailability.

**Conclusion:**

Integrating quercetin with nano‐based formulations presents a novel and promising approach for targeted skin cancer therapy. While preclinical results are encouraging, further experimental and clinical investigations are necessary to fully validate these findings and facilitate translation into clinical practice.

## 1 Introduction

Skin cancer is considered one of the most common cancers, with a mounting incidence that imposes remarkable pressure on healthcare systems [1]. This malignancy is related to the atypical growth of skin cells and is mainly developed in the areas subjected to ultraviolet (UV) rays [2]. In addition to UV light, several risk factors for skin cancer have been identified, such as impaired immune function, family history, arsenic compound exposure, and hereditary susceptibility [3, 4, 5]. Skin cancer can be categorized into two malignant types, comprising melanoma and non‐melanoma (squamous cell carcinoma [SCC] and basal cell carcinoma [BCC]). Conventional treatments like surgery, chemotherapy, and radiotherapy face limitations including side effects, resistance, and poor efficacy. Ergo, finding an alternative way to treat skin cancer with high efficiency and low challenge has been in the spotlight of scientists [6]. In the present era, natural compound‐based therapy is one of the popular therapeutic strategies to improve multiple human diseases, for instance, cancer, diabetes, neurodegenerative impairments, and cardiovascular diseases [7]. Natural compounds, particularly flavonoids, are chemical compounds with a natural origin obtainable from plants, fruits, fungi, etc., that possess a broad range of pharmacological benefits, such as anti‐inflammatory, antimicrobial, and anti‐cancer effects [8]. In this line, some popular natural compounds, particularly quercetin, a flavonoid compound extracted from onion, broccoli, and grapes, have been offered in skin cancer treatment in experimental studies [9, 10, 11]. Quercetin (C_15_H_10_O_7_), visible as a yellow powder, is one of the lipophilic natural agents with different pharmacological capacities, including anti‐inflammatory and antioxidative potentials, which have an indispensable role in making protection against skin damage induced by UV radiation [12]. Despite quercetin's potential to target skin cancer pathways, for instance, by inducing apoptosis and cell cycle arrest), challenges like low solubility and bioavailability necessitate harnessing alternative approaches, especially nano‐formulations [13]. Nowadays, several nano‐based drug delivery systems have been suggested for these natural compounds, like liposomes, nanoparticles (NPs), nanogels, polymers, and micelles, which effectually promote water solubility and bioavailability, therefore potentiating the efficacy while attenuating the toxic effects on normal cells [14]. Hence, studies regarding nano‐based herbal medicine against skin cancer are being conducted more and more. By this token, this narrative literature review aims to discuss the anti‐cancer potential of quercetin and its nano‐based formulations mechanistically in preclinical models as well as obstacles for clinical translation.

## 2 Method

In this narrative review, related documents with the status of “In press” and “Published” in the English language were evaluated qualitatively. Databases searched included Google Scholar, Scopus, PubMed, Web of Science, and Scientific Information Databases using keywords: “Skin cancer,” “Melanoma,” “Basal cell carcinoma,” “Squamous cell carcinoma,” “Quercetin,” “Nanotechnology,” and “Nano” until March 1, 2025. Initial searches retrieved 1247 records after duplicates were removed (total initial hits: 2156). Two independent investigators (MK and AE) screened titles and abstracts, excluding 1089 irrelevant records, followed by a full‐text review of 158 articles. Ultimately, 30 studies met the inclusion criteria: original and review articles focusing on quercetin and its nano‐based formulations in skin cancer (melanoma, BCC, SCC) across preclinical (in vitro/in vivo) or clinical settings; peer‐reviewed with adequate data. Exclusions comprised non‐relevant topics, insufficient information, or non‐peer‐reviewed sources. Disputes were resolved by a third reviewer (FRT). A summary of the included studies is given in Table 1.

**Table 1:** Number of studies reviewed in the field of quercetin effects on skin cancer.

Study type	Number	Key focus	Examples
In vitro	13	Apoptosis, proliferation inhibition in melanoma/SCC/BCC cells	B16, A375, SK‐MEL‐28
In vivo	1	Tumor reduction in mouse models (DMBA/croton oil)	Papilloma incidence delay
In vitro and in vivo	4	Inflammation and cell viability	IFN‐α, IFN‐β, STAT3
Nanoformulations (preclinical)	7	TiO2 NPs, nanoemulgels, PLGA‐TPGS enhancing bioavailability/efficacy	A431, B16‐F10 cells
Reviews/Mechanistic	5	Pathways (e.g., P53, STAT, IGF‐1, HIF‐1), ROS modulation	Comprehensive overviews

## 3 Skin Cancer Pathogenesis

### 3.1 SCC and BCC

**Figure 1:**
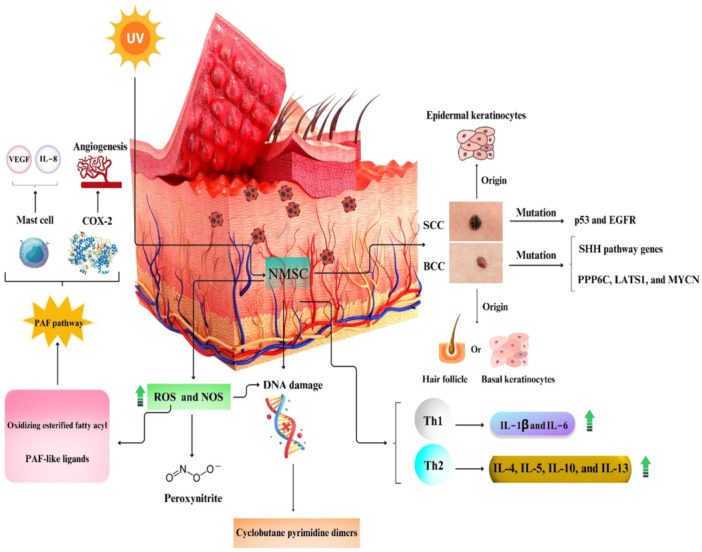
A summary of pathogenic mechanisms in non‐melanoma skin cancers (NMSCs).

SCC stems from epidermal keratinocytes with malignant proliferation properties [15]; however, BCC may originate from the hair follicle or basal keratinocytes of the interfollicular epidermis [16]. Environmental (especially UVB radiation) and genetic agents have a substantial role in the pathogenesis of non‐melanoma skin cancers (NMSCs) [17]. DNA damage resulting from UVB radiation leads to DNA structural changes, like cyclobutane pyrimidine dimers, causing a failure in DNA repair and alterations in the transcription and replication processes [18]. Besides DNA damage, UV can form reactive nitrogen and reactive oxygen species (ROS), leading to DNA oxidative damage. UV also raises cutaneous nitric oxide (NO) levels by stimulating NO synthase. Accordingly, increased ROS and NO levels lead to the production of peroxynitrite, an endogenous oxidant with high toxicity to DNA [19, 20]. Increased ROS production can oxidize esterified fatty acyl remnants and provide platelet‐activating factor‐like (PAF‐like) ligands. These events induce the PAF pathway, forming cyclooxygenase (COX) 2 and activating mast cells [21]. Elevated COX2 expression in NMSCs has been related to the promotion of angiogenesis and cancer cell invasion [22, 23, 24]. BCC incidence is accompanied by the overexpression of Th2 cytokines (e.g., IL‐4, IL‐5, and IL‐10) (Figure 1) [25, 26]. Other reports showed that the levels of Th1 cytokines, like IL‐1β and IL‐6, in BCC are higher than in SCC [26]. Following these occurrences, the upregulation of oncogenes, like glioma‐associated oncogene (GLI1), neuroblastoma RAS (NRAS), and Kirsten RAS (KRAS), and downregulation of tumor suppressor genes, such as neurogenic locus notch homolog protein 1 (Notch 1), have also been detected [18]. Sonic hedgehog (SHH) pathway is another key player in the pathophysiology of BCC [27, 28]. In this pathway, the binding of an SHH ligand to a transmembrane protein receptor (PTCH) suppresses the SMO, a proto‐oncogene, triggering transcription factors of the GLI [27]. In BCC, the lack of PTCH1 receptor activity is related to activation of the G protein‐coupled receptor SMO and Hedgehog signaling pathway [29]. Reportedly, about 85% of sporadic BCCs harbor mutations in SHH pathway genes, especially loss‐of‐function (LOF) mutations in PTCH. Moreover, BCC can carry mutations in other cancer‐associated genes, like PPP6C, LATS1, and RAS family members [30]. In SCC, p53 mutation and inactivation are considered key events of disease pathogenesis [29]. SCC development is regulated through a sophisticated interaction between different genes, comprising epidermal growth factor receptor (EGFR), cyclin‐dependent kinase inhibitor 2A (CDKN2A), TP53, and Notch 1, as well as the phosphoinositide 3‐kinase (PI3K)/Akt/mammalian target of rapamycin (mTOR) signaling pathways [31]. Besides, it has been confirmed that matrix metalloproteinase (MMP)−2 protein expression in SCC cases is higher than in BCC, addressing the vital role of MMP‐2 in the invasive behavior of skin tumors [32].

### 3.2 Melanoma

**Figure 2:**
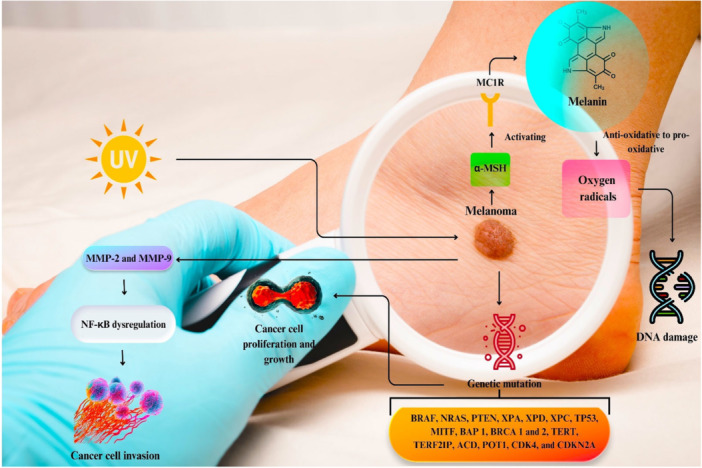
A summary of pathogenic mechanisms in melanoma.

Subjection to UV rays results in the secretion of α‐melanocyte‐stimulating hormone (α‐MSH), activating the melanocortin 1 receptor (MC1R) to produce melanin by melanogenesis [33]. Melanin has a compilation of pro‐oxidant and anti‐oxidant features. Under the influence of different etiological agents, like UV radiation, herbicides, and heavy metals, melanin can be converted from an anti‐oxidative to a pro‐oxidative element (Figure 2) [34, 35]. This phenomenon is the earliest pathogenic occurrence that activates carcinogenic processes. When melanin acts as a pro‐antioxidant agent, DNA damage is expected due to the elevation of intracellular oxygen radicals. The change in DNA sequence triggers diverse signaling pathways that finally cause unmanaged cell proliferation, immortalization, and dedifferentiation of particular cells [36]. Investigations have revealed that approximately 40%–50% of all melanoma patients represent an activated mutation in the BRAF proto‐oncogene encoding a serine/threonine protein kinase as a subset of the RAS/RAF/MEK/ERK kinase pathway, which potentiates cell proliferation and growth [37]. Mutations in the NRAS oncogene are also detected in 15%–20% of melanoma cases [38]. Alterations in the DNA sequence of other genes, for example, PTEN, XPA, and XPD, can indicate inherited melanoma [39]. Besides these processes, MMPs, especially MMP‐2 and ‐9, which have an inducer role in degrading extracellular matrix components, aid tumor cell infiltration and expansion. The upregulation of these proteins is linked with genetic changes and nuclear factor kappa B (NF‐κB) signaling pathway dysregulation [40]. According to reports, NF‐κB stimulates MMP‐9 overexpression by instigating osteopontin (OPN) as a protein associated with tumor microenvironment [41]. Also, the intragenic methylation process causes MMP‐9 overexpression in melanoma [42].

## 4 Overview of Quercetin

### 4.1 Origin and Source

Quercetin originates from the Latin term “quercetum,” meaning oak forest, and is one of the main flavonoids found in a broad range of plants and fruits. As a substantial component of the human diet, quercetin frequently exists chiefly as glycosylated derivatives in fruits (e.g., cherries, berries, apples, red grapes, and tomatoes), vegetables (garlic, onions, shallots, kale, and broccoli), and herb‐based beverages (red wine and herbal teas) [43, 44].

### 4.2 Physicochemical Properties

**Figure 3:**
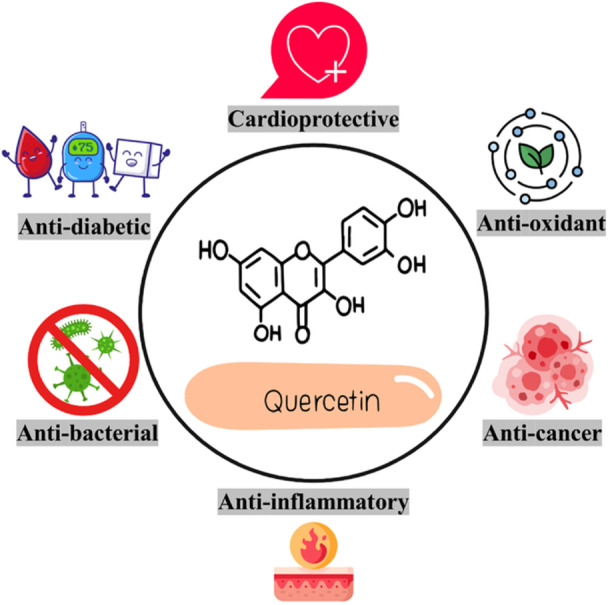
Quercetin and its biological benefits.

Quercetin is classified into a flavonoid class named 3‐hydroxyflavones. The molecular formula and weight of quercetin are C_15_H_10_O_7_ and 302.24 g/mol, respectively. Its chemical structure (Figure 3) consists of hydroxyl groups at locations of C‐3′ and C‐4′ of the B‐ring, C‐5 and C‐7 of the A‐ring, and C‐3 of the C‐ring, which has a defining role in the number of derivatives and pharmacological capacities [45]. Several derivatives of quercetin exist, including glycosides and methylated derivatives, as well as the less common prenyl and sulfate substituents [46]. Quercetin is commonly conjugated with rutinose and glucose in glycosidic forms, and the hydroxyl group at C‐3 is the most common glycosylation site [47]. Generally, quercetin is known as a lipophilic agent with low bioavailability and solubility and a 3.5 h‐half‐life [43]. Its estimated aqueous solubility is 1 μg/mL, while it is 28.9 μg/mL in intestinal fluid and 5.5 μg/mL in gastric fluid [48]. It is worth mentioning that quercetin solubility is different among its derivatives and indeed attributable to the kind of substituents. For example, glycosylated forms of quercetin are capable of elevating its hydrophilicity [49]. The primary site for absorbing this flavonol is the small intestine, though it can also be absorbed in the stomach. The absorption is mediated by passive diffusion for the aglycone or the sodium‐glucose cotransporter‐1 (SGLT1) for glycosides [50]. One of the main pharmacological challenges of quercetin is its poor bioavailability in light of its absorption profile, chemical stability, and low water solubility [51]. It is stated that only 20% of the orally prescribed dose of quercetin is absorbable. The bioavailability of quercetin is remarkably limited by intestinal metabolism [52]. In the first stage, it is metabolized by cytochrome P450 enzymes. In the second stage, conjugation occurs in the small intestine through glucuronidation, methylation, and sulfation [53]. Subsequently, the metabolites are released into the lymphatic circulation and bloodstream. When quercetin is taken orally, it is excreted through feces and urine; however, some metabolites are also excreted via the gallbladder [54].

### 4.3 Pharmacological and Biological Capacities

Quercetin is known as a safe flavonoid with a broad range of pharmacological and biological features [55]. These beneficial features include anti‐cancer [56], anti‐inflammatory [57], antioxidant [58], and immunomodulatory [59] influences. Moreover, numerous studies have indicated its anti‐fungal [55], anti‐viral [47], anti‐bacterial [60], anti‐diabetic [61], hepatoprotective [62], cardioprotective [63], neuroprotective [64], and renoprotective [65] capacities by triggering different signaling pathways. This evidence highlights the health benefits of quercetin in the current medical era and encourages researchers to seek other pharmacological aspects.

### 4.4 Quercetin and Pharmacological Concerns

Although numerous studies have shown that quercetin possesses a tremendous therapeutic potential for treating health‐threatening diseases, some concerns need to be considered. Low bioavailability, poor permeability, poor water solubility, instability, poor absorption, and retention in cells are among the main pharmacological challenges of quercetin therapy [66, 67]. Moreover, poor tumor‐targeting biodistribution, short half‐life, rapid systemic elimination, rapid metabolism, poor absorption, and limited skin permeation are other pharmacological problems that limit its extensive use in the clinic for cancer therapy via oral or topical routes [68, 69]. Alongside these concerns, the presence of some side effects and drug interactions motivates scientists to find alternative approaches to cancer treatment [70]. A number of toxicity‐related studies have demonstrated organ toxicity following high‐dose quercetin administration in animal models, particularly liver toxicity and nephrotoxicity [70, 71]. Routinely, oral consumption of quercetin in humans appears to be safe and well‐tolerated. Many clinical trials have confirmed the safety of quercetin with doses up to 1 g/day [72]. The emergence of non‐serious side effects, like headache, mild tingling of the extremities, and mild stomach discomfort, has been reported after quercetin intake with doses up to 5 g daily [73]. On the other hand, some animal studies have reported adverse effects, for example, decreased body weights, the appearance of non‐neoplastic hyperplastic polyps of the cecum, and the existence of calcium oxalate crystals in the urine, following a high‐dose quercetin administration (1900 and 2100 mg/kg). Furthermore, yellow‐brown pigmentation in the small intestine and glandular stomach following quercetin therapy in high doses was observed, which may be caused by the yellow color of quercetin or by one of its metabolites [74, 75, 76]. Animal and human investigations have also displayed that quercetin can change the bioavailability of diverse drugs, leading to an increased likelihood of adverse drug effects. Some animal projects have divulged that quercetin is able to elevate the drug bioavailability of several anti‐cancer drugs (e.g., doxorubicin, paclitaxel, and irinotecan), ranolazine (an anti‐anginal drug), tamoxifen (an antiestrogen drug), valsartan (an anti‐hypertensive drug), and digoxin (a drug against heart failure) [77, 78, 79, 80, 81, 82, 83]. In human studies, drug bioavailabilities were decreased in talinolol (antihypertensive drug) and midazolam (sedative) and increased in fexofenadine (antihistamine drug) and pravastatin (cholesterol‐lowering drug) [84, 85, 86, 87]. Moreover, one study has stated that quercetin (25–100 mg/kg orally) decreases dose‐dependent catalepsy caused by intraperitoneal application of reserpine, α‐methyl‐p‐tyrosine, or perphenazine in rats [88]. Considering these pharmacological concerns, an alternative method should be applied that addresses these issues and enhances its efficacy against malignancies, like skin cancer.

## 5 Quercetin Against Skin Cancer

**Figure 4:**
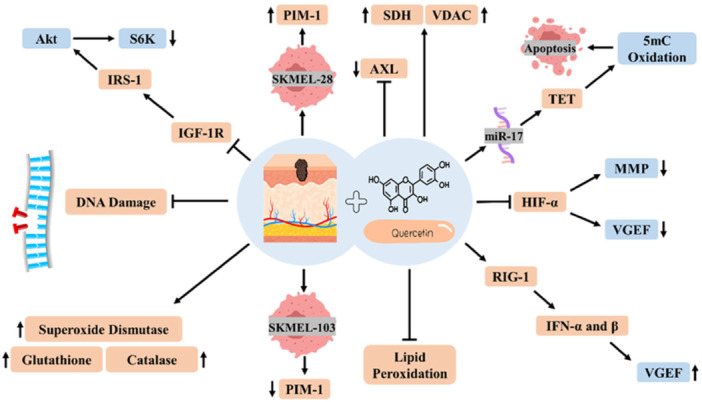
Quercetin can target skin cancer through diverse cellular and molecular mechanisms.

Several preclinical studies have underscored the anti‐cancer potential of quercetin for skin cancer through different mechanisms and signaling pathways (Table 2). To shed more light on this subject, an in vitro study claimed that the anti‐melanoma effects of quercetin are thanks to inhibiting tyrosine kinase receptors (PIM‐1 and AXL) and elevating the expression of mitochondrial proteins (SDH and VDAC). In this investigation, quercetin could suppress AXL in two cell lines of SKMEL‐28 and SKMEL‐103; however, PIM‐1 expression was increased in SKMEL‐28 cells and decreased in SKMEL‐103 cells following the treatment. Also, a decrease in hypoxia‐inducible factor‐1α (HIF‐1α) expression was observed in both cell lines [89]. HIF‐1α has tumor‐enhancing impacts in melanoma by potentiating VEGF secretion and inducing MMPs [90]. One of the interesting outcomes of this work was that quercetin is more efficient than kinase suppressors like erlotinib, imatinib, U0126, and temsirolimus [89]. Another in vitro work scrutinized the anti‐carcinoma effects of this plant‐derived flavonoid on B16 murine melanoma cells and showed a 75% decrease in cell viability of B16 cells 6–48 h after quercetin therapy (50 μg/mL) [11]. The rate of cell viability reduction in quercetin‐treated cells was more than in cells exposed to etoposide, a chemotherapy drug. This flavonoid could also decrease the proliferative capacity of B16 cells more efficiently than etoposide. Moreover, quercetin decreased the number of cells in the stages of S and G2/M of the cell cycle. This happening can be justified by apoptosis induction, as evidenced by a flow cytometry test of Annexin V+ cells, a key protein binding to phosphatidylserine (PS), usable to detect apoptotic cells [11]. Ali et al. [61] inspected the chemopreventive capacity of quercetin in a mouse model of skin carcinoma established by topical administration of croton oil and 7, 12‐dimethyl Benz (a) anthracene (DMBA). Following disease establishment, quercetin (200 and 400 mg/kg for 16 weeks, orally) was prescribed [91]. The application of croton oil and DMBA in mice results in papilloma formation as well as liver damage, as evidenced by an increase in the levels of bilirubin, alkaline phosphatase (ALP), serum glutamic pyruvic transaminase (SGPT), and serum glutamic oxaloacetic transaminase (SGOT) [92]. The results of this experimental work pointed out the capability of this flavonoid to reduce papilloma numbers and the mentioned liver biochemical parameters. This natural product could also elevate the antioxidant defense system by elevating the levels of antioxidant enzymes (glutathione, catalase, and superoxide dismutase), attenuating lipid peroxidation, and diminishing DNA damage in animal models of skin carcinoma [91]. Jung et al. [93] investigated quercetin's influence on suppression of the insulin‐like growth factor 1 (IGF‐1) signaling pathway in transgenic mice, whose IGF‐1 expression level in their skin epidermis is considerably high [93]. They found that administration of a diet rich in quercetin (0.02% wt) postpones skin tumor incidence by 14 days and reduces the multiplicity of skin tumors by 35% in comparison with the control group treated with tetradecanoyl phorbol‐13‐acetate (TPA), a carcinogenic agent for mouse skin. Quercetin supplementation was also able to reduce TPA‐conferred skin hyperplasia in transgenic mice. To deliberate the action mechanisms of quercetin in suppressing skin tumor development in teratogenic mice, the phosphorylation of proteins related to the IGF‐1 signaling pathway in the MT1/2 cells (a papilloma cell line) was monitored after quercetin treatment (10–50 µM). In the end, quercetin repressed IGF‐1‐ caused phosphorylation of the IGF‐1 receptor (IGF‐1R), S6K, Akt, and insulin receptor substrate (IRS)−1 dose‐dependently, indicating IGF‐1 signaling suppression [93]. Lately, ten‐eleven translocation (TET) protein expression was investigated in an in vitro and in vivo study (2023) to examine the suppressive effects of quercetin on uveal melanoma cells [94]. TET protein catalyzes 5‐methylcytosine (5mC) oxidation and mediates apoptosis induction in some malignancies [95, 96]. The authors observed increased TET1 expression after quercetin therapy in melanoma cells, including B16, SK‐MEL‐1, and OCM‐1. Bioinformatic analyses also demonstrated that TET1 is the miR‐17 target gene. Consequently, this research team inferred that miR‐17 affects cell growth and apoptosis in malignant cells by regulating TET1 expression (Figure 4). These reports were consistent with the in vivo part of this study in which nude mice harboring OCM‐1 cells were treated with 100 mg/kg quercetin (for 21 days). In animal models of melanoma, quercetin repressed tumor growth compared with the untreated group [94]. Another preclinical study indicated the suppression of cell proliferation and induction of apoptosis as a result of quercetin therapy in A375 (1–60 μM) and B16 cells (0.1–50 μM). Molecular analyses confirmed that the utilization of this natural compound leads to the overexpression of interferon (IFN)‐α and ‐β by triggering retinoic acid inducible gene‐I (RIG‐I) promoter in melanoma cells (B16 cells). RIG‐I is a cytosolic PRR that identifies 5′‑triphosphate RNA produced by viral RNA polymerases and possibly promotes anti‐cancer impacts by triggering signal transduction and activator of transcription 1 (STAT1) in paracrine and autocrine signaling pathways. In vivo results of this preclinical work unveiled the attenuation of mouse melanoma growth, as shown by decreasing tumor volume, weight, and growth curve, in mice harboring B16 cells following intragastric application of quercetin (75 mg/kg/day) [97]. Other anti‐cancer capacities of quercetin in skin cancer have been summarized in Table 2.

**Table 2:** The therapeutic effectiveness of quercetin against skin cancer.

Dose/concentration (s)	Target (s)	Effect/mechanism (s)	Major finding (s)	In vivo/In vitro	Species	Cell line (s)	Reference
100 mg/kg and 50 and 100 μM	TET1 and miR‐17	Stimulating apoptosis and repressing cell migration and invasion	Quercetin induces TET1‐caused apoptosis Quercetin elevates the expression of TET1 (a target gene of microRNA‐17)	In vivo and in vitro	Nude mice	B16, SK‐MEL‐1, and OCM‐1	[94]
50 and 100 µM	SDH, VDAC, PIM‐1, AXL, HIF‐1α, Akt, HSP90, and HSP70	Attenuating the expression of tyrosine kinase receptors, elevating the expression of mitochondrial proteins, and decreasing cell viability	Quercetin decreases the cell viability of SKMEL‐103 cells without affecting SKMEL‐28 cells The anti‐cancer effects of quercetin against SKMEL‐103 cells are stronger than kinase inhibitors (e.g., Erlotinib, Temsirolimus, and Imatinib)	In vitro	—	SKMEL‐28 and SKMEL‐103	[89]
0–50 µM	Caspase 3, Nrf2, NF‐κB, and MAPK	Promoting or attenuating ROS production and cell growth dose dependently	Quercetin at high concentrations decreases the growth of melanoma cells. In contrast, at low concentrations, it has the inverse impact on metastatic melanoma spheroids but not on the non‐metastatic cell line High quercetin concentrations (> 12.5 µM) reduce cell viability, while low concentrations (< 6.3 µM) promote cell proliferation and spheroid size	In vitro	—	MCM DLN, MCM 1G, and 1205Lu	[98]
0.1–100 mg/mL	—	Stimulating apoptosis, repressing cell proliferation, and decreasing cell viability	Quercetin therapy leads to a 75% decrease in the viability of B16 melanoma cells The decrease in cancer cell viability was similar to or greater than that observed with etoposide, a well‐known chemotherapeutic agent	In vitro	—	B16	[11]
3, 15, 75 mg/kg and 0.1–10 µM	IFN‐α, IFN‐β, and RIG‐I	Repressing tumor growth, inducing apoptosis, and inhibiting cell proliferation	Quercetin serves as a RIG‐I agonist Quercetin enhances the expression of IFN‐α and IFN‐β by triggering the RIG‐I promoter in B16 cells	In vivo and in vitro	Wild‐type C57BL/6J male mice	A375 and B16	[97]
50 and 100 mg/kg and 0–200 µM	Phospho‐JNK, Bax, phospho‐ERK1/2, phospho‐p38, Bcl‐2, and cleaved poly‐ADP ribose polymerase	Stimulating apoptosis, attenuating cell viability and proliferation, and repressing tumor growth	Quercetin considerably reduces the proliferation and viability of A375SM cells in a concentration‐dependent way without affecting A375P cells	In vivo and in vitro	Female BALB/c nude mice	A375P and A375SM	[99]
25–150 µM	—	Regulating mitochondrial and glycolytic mechanisms for ATP generation by reducing indices related to oxygen consumption rate and extracellular acidification rate	Seventy‐two hours of therapy with quercetin reduces all oxygen consumption rate‐related indicators (i.e., proton leak, basal respiration, maximal respiration, reserve capacity, and ATP turnover)	In vitro	—	B164A5	[100]
25 and 50 µM	DR4, DR5, and FLIP	Inducing apoptosis	Quercetin serves by elevating the expression of rhTRAIL‐binding receptors DR4 and DR5 on cancer cells and by enhancing the proteasome‐associated degradation of the anti‐apoptotic protein FLIP	In vitro	—	WM164 and MeWo	[101]
5–40 μM	PARP‐1, caspase‐3, and caspase‐8	Inducing apoptosis, decreasing cell viability, repressing cell growth, arresting cell cycle	Quercetin enhances cell death in B16F10 cells exposed to UVB by increasing reactive oxygen species (ROS) production, depolarizing the mitochondrial membrane potential (ΔΨM), regulating the antioxidant defense response, and influencing calcium homeostasis	In vitro	—	B16F10	[102]
20–80 μM	c‐Met, HGF, Gab1 (GRB2‐associated‐binding protein 1), PAK (p21‐activated kinases), and FAK (Focal Adhesion Kinase)	Repressing cell migration and invasion	Quercetin inhibits melanoma cell migration and invasion dose‐dependently once induced by HGF PAK or FAK upregulation dramatically decreases the suppressive influence of quercetin on melanoma cell migration	In vitro	—	A375, A2058, sk‐mel‐2, and MeWo	[103]
200 and 400 mg/kg	—	Decreasing tumor size, DNA damage, the number of papillomas, the serum levels of bilirubin, glutamate pyruvate transaminase, alkaline phosphatase, and glutamate oxalate transaminase, elevating the levels of catalase, superoxide dismutase, and glutathione, and repressing the level of lipid peroxides	Quercetin significantly reduces DNA damage in treated mice in comparison with mice treated with croton oil and 7, 12‐dimethyl benz (a) anthracene (DMBA)	In vivo	Swiss albino mice	—	[91]
100 mg/kg and 0–60 µM	STAT3, VEGF, MMP‐2, MMP‐9, and Mcl‐1	Stimulating apoptosis, decreasing cell viability, repressing cell proliferation, migration, and invasion, and tumor growth	Quercetin represses the STAT3 signaling pathway by regulating STAT3 phosphorylation and decreasing STAT3 nuclear localization As a result, transcription activity of the STAT3 is suppressed, and STAT3 targeted genes, including VEGF, Mcl‐1, MMP‐2, and MMP‐9, are downregulated	In vivo and in vitro	Male nu/nu BALB/c mice	A2058 and A375	[104]
0–100 µM	DNApk and ΔNp73	Inducing apoptosis	Quercetin combined with Temozolomide eliminates drug insensitivity and considerably promotes apoptosis compared to either treatment separately Quercetin therapy confers ΔNp73 redistribution into the cytoplasm and nucleus, which is linked with elevated p53 transcriptional function	In vitro	—	SK Mel 5, SK Mel 28, and DB‐1	[105]
5–75 µM	P53 and Bax	Increasing ROS levels, decreasing GSH levels, and inducing apoptosis	In tyrosinase clones, quercetin reduces bioreduction potential and elevates ROS formation more significantly compared to control cells The stimulation of apoptosis confered by quercetin therapy was mediated by the p53/Bax pathway	In vitro	—	DB‐1	[106]
20, 50, and 100 µM	—	Repressing DNA damage and scavenging free radicals	DNA protective capacity of quercetin at the concentrations of 20 and 100 μM against free radicals in human melanoma cells (HMB‐2) is 40% and 80%, respectively	In vitro	—	HMB‐2	[107]
0.5–20 µM	Tyrosinase	Promoting melanogenesis	Quercetin therapy (1 or 20 μM) for 7 days increases melatonin amount compared with controls Cycloheximide or actinomycin‐D can block Tyrosinase activation conferred by quercetin	In vitro	—	HMVII	[108]
3.3 × 10^−1^ mM	PKC and MMP‐2 and ‐9	Repressing cell invasion	Quercetin reduces the gelatinolytic function of pro‐MMP‐9 in a dose‐dependent manner Quercetin shows a dose‐dependent ability to antagonize the increases in gelatinolytic function of pro‐MMP‐9 conferred by free fatty acids and phorbol‐12,13‐dibutyrate (PDB)	In vitro	—	B16‐BL6	[109]
10^−6^–10^−4^ g/mL	Bcl‐2	Repressing cell mobility and invasion, arresting cell cycle, attenuating cell proliferation and migration, and inducing apoptosis	Quercetin retards the cell cycle in the S and G2–M stages of the cell cycle in a dose‐dependent manner Quercetin significantly suppresses the expression of Bcl‐2 (anti‐apoptotic protein) without a marked effect on Bcl‐XL (another anti‐apoptotic protein)	In vitro	—	B16‐BL6	[110]

Quercetin exhibits biphasic effects, acting as an antioxidant at low concentrations (< 40 μM) to protect cells but switching to pro‐oxidant at higher doses (40–100 μM), generating ROS, o‐quinone adducts, and depleting glutathione (GSH), which selectively induces apoptosis in cancer cells like melanoma while sparing normal cells. In skin cancer models, this pro‐oxidant activity enhances cytotoxicity in UVB‐irradiated B16F10 melanoma cells but may potentiate UV‐induced signaling like c‐Fos in some contexts. These dose‐dependent effects necessitate careful optimization to avoid unintended toxicity, such as organ damage observed in high‐dose animal studies [111].

## 6 Nano‐Based Formulations of Quercetin and Skin Cancer

### 6.1 Titanium Dioxide NPs

**Figure 5:**
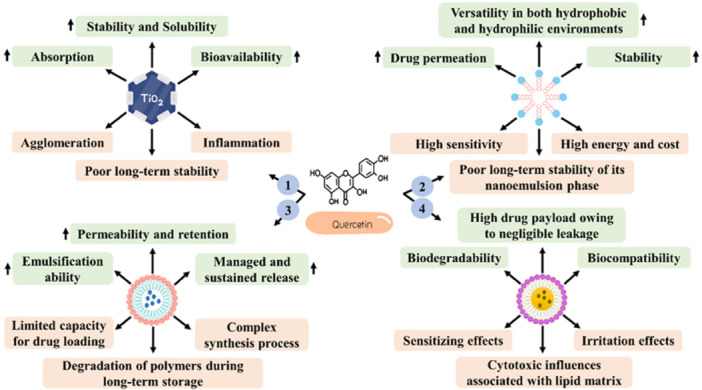
Nano‐based formulations of quercetin can potentiate the therapeutic effectiveness of quercetin in the fight against skin cancer. 1, Titanium dioxide nanoparticles; 2, Nanoemulsion; 3, Nanostructured Lipid carrier; 4, PLGA‐TPGS nanoparticles.

Recently, titanium dioxide (TiO_2_) (Figure 5) has been known as a favorable mineral in various fields thanks to its unique chemical and physical features. Currently, there are two crystal structures of TiO_2_, including anatase, with highly photocatalytic, and rutile; the second form (rutile) is less photocatalytic and more stable, and chemically is assumed inert [112]. Morphologically, TiO_2_ can be synthesized in the form of spherical particles, nanotubes, and nanowires/nanorods [113]. TiO_2_ possesses diverse advantages, such as being non‐toxic, safe, chemically and thermally stable, biocompatible, having outstanding optical properties, antimicrobial features, and the capability to absorb and scatter UV radiation, making it an interesting element in diverse daily products, such as skin care creams, toothpaste, and food colorants [114]. However, there are some limitations regarding these NPs. The possible toxicity of TiO_2_ NPs, especially once ingested or inhaled, has been one of the main concerns of these NPs because their application may give rise to inflammation and oxidative stress [115]. Other problems related to TiO_2_ NPs comprise poor photocatalytic performance under visible light, the tendency to agglomerate, causing reduced surface area and weak functionality in drug delivery‐related purposes, and high cost for synthesizing high‐quality TiO_2_ NPs [116, 117, 118].

It has been addressed that TiO_2_ NPs are one of the suitable choices to encapsulate quercetin in order to elevate its stability and solubility in aqueous media, leading to its bioavailability and absorption enhancement [119]. Also, these NPs are capable of safeguarding quercetin from enzymatic degradation and some environmental factors, for example, pH and light, aiding in its stability in the time of reaching the desired region [120, 121]. Moreover, these metallic NPs can overcome the pharmacological limitation of quercetin by minimizing systemic toxicity, reducing the need for high‐dose quercetin, and releasing it in a sustained and controlled manner [122, 123, 124].

Having considered the anti‐cancer capacity of quercetin against skin cancer, which was discussed above, and the biochemical benefits of these NPS, the anti‐skin cancer effects of TiO_2_NPs loaded with quercetin have been investigated [125]. In this line, Chahardoli et al. [125] have fabricated and characterized TiO_2_NPs loaded with quercetin (TiO_2_NPs‐Que) and assessed their biological features, such as cytotoxicity, anti‐inflammatory, and anti‐hemolytic influences, against melanoma cell lines (A375) in vitro. The characterization methods, including Transmission/Field‐Emission Scanning Electron Microscopy (TEM/FE‐SEM) and X‐ray diffraction (XRD), indicated that the designed TiO_2_NPs‐Que had a spherical shape, belonged to the rutile phase, and had a size range of 7.3–39 nm. TiO_2_NPs‐Que did not display hemolytic influences. They demonstrated 95.3% stabilization activity of red blood cell (RBC) membranes and 82.6% suppression of bovine serum albumin (BSA) denaturation, like a standard drug, validating their anti‐inflammatory impacts. Cytotoxicity‐related data indicated the toxicity of the synthesized nano‐based formulation of quercetin on A375 without considerable effects on normal skin fibroblast cells [125]. The reason behind this result can be related to its potential to increase ROS formation, causing cancer cell death [125, 126]. Ergo, it is suggested that TiO_2_NPs‐Que can exert anti‐cancer effects against skin cancer by reducing cell viability and inflammatory reactions and increasing ROS production, resulting in cell death. Nevertheless, additional experimental evidence is needed to support this theory.

Nano‐based systems like TiO_2_ NPs and nanoemulgels improve quercetin's delivery but introduce NP‐specific risks, including inflammation, oxidative stress, agglomeration, poor long‐term stability, and potential organ accumulation, particularly with TiO_2_ inhalation or ingestion. Contradictory preclinical data highlight variable efficacy across formulations, with some showing selective cancer cell toxicity but others raising concerns over skin irritation from surfactants or scalability issues. Balanced assessment reveals that while nano‐quercetin enhances bioavailability, unresolved safety profiles limit clinical readiness [127].

### 6.2 Nanoemulgel

Structurally, nanoemulgels are known as hybrid colloidal systems established by nanoscale oil droplets distributed in a gel‐based aqueous matrix, putting together the features of both hydrogels and nanoemulsions [128]. A nanoemulsion is also a liquid formulation with a high stability pharmacokinetically, featuring droplet diameters ranging from 10 to 100 nanometers [129]. These formulations have several superiorities over traditional options, comprising enhanced penetration to deeper skin layers, promoted drug solubility, and a reduced dosage, resulting in minimized dose‐associated side effects [129].

Thanks to nanoemulgel, the easy incorporation of lipophilic drugs, as well as the potentiation of permeability of the combined drugs, is conceivable owing to disseminated droplets related to the nanoemulsion phase. Accordingly, the pharmacodynamic and pharmacokinetic parameters of lipophilic drugs are considerably enhanced [130].

Overall, the current evidence suggests that nanoemulgel offers a variety of benefits. These include enhanced drug permeation, improved stability, and versatility in both hydrophobic and hydrophilic environments [131, 132, 133]. Additionally, nanoemulgel aids in the targeted delivery of drugs, provides controlled release, and typically causes fewer side effects than traditional formulations, making it less irritating to the skin [134].

Despite these, some challenges are limiting the wide utilization of nanogel‐based drug delivery systems. One of these issues is related to the stability of the gel phase. Most of the gelling agents have a high sensitivity to temperature and pH changes [130]. Other problems comprise the need for high energy and cost to nanoemulsion preparation, the restricted long‐term stability of its nanoemulsion phase, and the possibility of incidence of dermatitis and skin irritation following its application due to a high amount of surfactant [135, 136, 137].

One of the nano‐based strategies for improving pharmacological challenges and enhancing the anti‐cancer effects of quercetin against skin cancer has been developed based on nanoemulgel [69]. Nanoemulgel can overcome the restriction of quercetin in cancer therapy by promoting stability and bioavailability, improving release capacity, decreasing quercetin therapy‐related side effects, and boosting skin penetration for topical applications [69, 138].

In this regard, Chitkara and colleagues designed a stable quercetin nanoemulgel by adding Carbopol 940 (a gelling agent) through an ultrasonication emulsification technique and appraised its toxicity in vivo and effectiveness on human skin cancer cells (A431) in vitro. The characterization of the fabricated nanoemulgel was carried out using SEM, Fourier Transform‐Infrared (FT‐IR) spectroscopy, assessment of zeta potential and entrapment efficiency (%EE), and particle size analysis. Moreover, the designed quercetin‐loaded nanoemulgel was characterized by determining pH and viscosity and evaluating the texture profile. The nanoemulgel had a nanosize (173.1 ± 1.2 nm) along with decreased polydispersity index (0.353 ± 0.13), zeta potential (−36.1 ± 5.9 mV), and suitable %EE (90.26%). The toxicity assessments showed that the nanoemulgel is well‐tolerated and safe, given that no organ toxicity or skin irritation was observed in Wistar rats. Moreover, the nanoformulation was safe for topical utilization. In vitro reports indicated that quercetin nanoemulsion had a higher releasing capacity compared with the pure suspension of quercetin (85% vs. 26%) in vitro in light of its small globular size, giving rise to large surface area for promoting hydrophobic drug solubility and elevating diffusion rate. The outcomes also revealed that the nanoemulgel form of quercetin had higher cytotoxicity to A431 cells than the pure quercetin, likely because of the usage of olive oil in this nanoformulation, which possesses anti‐cancer effects on skin carcinomas [69]. The anti‐cancer potential of olive oil has been addressed through different mechanisms, such as apoptosis induction and its effect on tumor proliferation and metabolism [139]. Another experimental study examined the impacts of oral application of a nanosized emulsion comprising quercetin on the cytotoxicity of melanoma in vitro (B16‐F10 cells) and in vivo (mice bearing B16‐F1O cells) (Table 3) [140]. The nanosized emulsion was designed through hot solvent diffusion related to the phase inversion temperature techniques. In vitro outcomes implicated a decrease in cell survival dose‐dependently for pure quercetin and nanoemulsion of quercetin. In in vivo assessments, quercetin was administered either as a pure drug or in a colloidal dispersion at a dose of 5 mg/kg two times per week for 17 days orally. Results pointed out that both pure quercetin and quercetin nanoemulsion reduced tumor growth; however, the effect was more significant in animals treated with quercetin nanoemulsion. In addition, there was no evidence showing hepatic or renal toxicities following the administration of quercetin in either its pure or nanoformulated forms [140]. These reports highlighted that the oral bioavailability of quercetin improved once the compound was incorporated into the oily phase of a nanosized emulsion, offering a potential application in skin cancer treatment. Moreover, the anti‐skin cancer effects of quercetin nanoemulgel were emphasized by addressing its role in attenuating cell viability and tumor growth.

**Table 3:** The therapeutic capacities of nano‐based formulations of quercetin against skin cancer.

Type of nanoformulation	Dose/concentration (s)	Effect/mechanism (s)	In vivo/In vitro/Ex vivo	Species	Cell line	Reference
Quercetin loaded with titanium dioxide NPs	6.25–100 μg/mL	Decreasing cell viability, inducing ROS formation and cell death, and attenuating inflammation	In vitro	—	A375	[125]
Transliposomal gel loaded with quercetin	2.5–50 µM	Exerting cytotoxic effects on cancer cells	In vitro	—	B16F10	[141]
Quercetin and curcumin loaded with optimized Mesoporous Silica NPs	0–200 µM	Exerting cytotoxic effects on cancer cells	In vitro	—	A375	[142]
Nanoemulsion loaded with quercetin	7.80–1000 μg/mL	Exerting cytotoxic effects on cancer cells	In vivo and in vitro	Male Wistar rats	A431	[69]
Nanosized emulsion comprising quercetin	5 mg kg	Decreasing cell viability and tumor growth	In vivo and in vitro	C57BL/6 mice	B16‐F10	[140]
Nanoformulated lipid carrier gel of quercetin and resveratrol	12.5–200 µM	Exerting cytotoxic effects on cancer cells dose dependently	In vitro and ex vivo	—	A431	[143]
Nanogel of quercetin and titanium dioxide	Qu (0.12%) + TiO2 (5%) nanogel	Decreasing tumor volume, cell proliferation, and inflammation	In vivo	SKH‐1 mice	—	[144]

### 6.3 Poly (d,l‐Lactide Co‐Glycolide) (PLGA) NPs Emulsified With TPGS

Nowadays, NPs obtained by biodegradable copolymers like Tocopheryl polyethylene glycol 1000 succinate (TPGS)‐conjugated Poly (d,L‐lactide co‐glycolide) (PLGA) (PLGA‐TPGS) hold promise in drug delivery systems [145]. Poly (d,L‐lactide co‐glycolide) (PLGA) is a biodegradable polymer approved by the Food and Drug Administration (FDA) with acceptable biodegradability and biocompatibility; thus, the PLGA‐based NPs can be a good choice for carrying hydrophobic or hydrophilic drugs [146, 147]. PLGA‐TPGS NPs are composed of three main subsets, including PLGA Core, TPGS Shell, and drug payload (a drug encapsulated in the PLGA core) [148]. The core of these NPs is made from PLGA, which is a copolymer of glycolic acid and lactic acid [149]. TPGS, a natural vitamin E derivative soluble in water, acts as a shell around the PLGA and is extensively applied as a bioavailability, solubilizer, and emulsifier of hydrophobic drugs [150].

PLGA‐TPGS NPs possess wide applications due to their improved cellular uptake, high emulsification ability, enhanced biocompatibility and biodegradability, safety, stability, managed and sustained release, and enhanced permeability and retention (EPR) influence [150, 151, 152]. On the contrary, complex synthesis process, limited capacity for drug loading, and degradation of polymers during long‐term storage are the main challenges related to PLGA‐TPGS NPs.

Interestingly, PLGA‐TPGS NPs loaded with quercetin have been recommended by Zhu and co‐workers to combat skin damage arising from UVB [153]. In this study, PLGA‐TPGS NPs were synthesized via the nanoprecipitation method. The NPs had a core‐shell structure, with hydrophobic PLGA providing the core encapsulating the water‐insoluble quercetin and a TPGS segment serving as the hydrophilic stabilization shell. The encapsulation efficiency and drug content of quercetin‐loaded PLGA‐TPGS NPs were almost 81.7% and 8.62%, respectively, revealing a good performance for a suitable drug delivery system. This research team found that quercetin encapsulated with PLGA‐TPGS NPs (100–300 μL/mL) can solve the problem related to the poor hydrophilicity of quercetin and exert anti‐UVB impacts by suppressing inflammatory mediators (NF‐kB and COX‐2) and mitigating histopathological features caused by UVB irradiation in human keratinocyte cell lines (Hacat cell line). Thanks to this nanocarrier, quercetin was considerably more sustainable in the dermis/epidermis than in the stratum corneum, indicating the potential ability of LGA‐TPGS NPs for skin delivery of quercetin. The histopathological analyses of this work manifested that quercetin‐loaded NPs attenuated the UVB‐conferred erratic arrangement of collagen fiber, reduced the thickness of dermal collagen fiber, and increased epidermal thickness [153]. Thus, PLGA‐TPGS NPs loaded with quercetin can, on the one hand, surmount the pharmacological obstacles of quercetin, mainly by enhancing its aqueous solubility, sustained and controlled release, and targeted delivery to selected regions. On the other hand, they can have a protective role against skin damage arising from UVB radiation, mainly by modulating histopathological, immunological, and inflammatory factors.

### 6.4 Nanoformulated Lipid Carrier

Nanoformulated lipid carriers are new drug delivery systems that contain physiological and biodegradable solid and liquid lipids, which have been inspected for targeted transportation of drugs to the skin [154]. These structures are second‐generation lipid nano‐transporters that are fabricated by a solid lipid matrix combined with liquid lipids [155]. This type of carrier, unlike emulsions, can efficiently immobilize drugs and prevent particle coalescence due to the solid matrix [155]. Nanoformulated lipid carriers have attractive biomaterial characterizations, such as small size, biodegradability, and biocompatibility, EPR impact, the existence of a disordered crystal structure, stability, and high drug payload owing to negligible leakage, and the feasibility of formulating over liposomes and solid lipid nanoparticles (SLN) [156, 157]. The small size of these nanoformulations ensures close contact with the skin, increasing the amount of drug that penetrates the skin [158]. The usage of nanoformulated lipid carrier gel topically provides a lipid film on the skin that limits trans‐epidermal water loss (TEWL), promotes skin moisture, and keeps it hydrated [159, 160]. The limitations of the application of nanoformulated lipid carriers include cytotoxic influences associated with lipid matrix, irritation and sensitizing effects of surfactants, and issues related to the stability of lipids [161].

Imran et al. [143] proposed the topical application of a nanoformulated lipid carrier gel containing quercetin and resveratrol in skin cancer to improve their distribution in epidermal and dermal layers in vitro and ex vivo. This nano‐based formulation included a surfactant (Cremophor RH40) and lipid binary mixture (1.0% w/w). The mean particle size and polydispersity index of the nanoformulated lipid carrier were 191 nm ± 5.20 and 0.33 ± 0.01, respectively, showing dispersion homogeneity and competency of the formulated nanogel for dermal delivery. The cytotoxic evaluations of various concentrations (12.5–200 μM) of the gel in A431 cell lines were carried out using the MTT method. The permeability and flux coefficients of quercetin and resveratrol from nanoformulated lipid carrier gel were 3.70 μg/cm²/h, 14.09 μg/cm²/h, and 4.69 × 10^−2^ cm/h, and 7.21 × 10^−2^ cm/h, respectively, suggesting their good potential for deeper layers of the skin. This formulation also had a high entrapment effectiveness and drug loading due to the solubility of these two natural compounds in lipids and the disorganized crystal structure of the nanogel. IC50 values for conventional gel, which was obtained using Carbopol P934 (a polymer of acrylic acid cross‐linked with polyalkenyl alcohols), and nanoformulated gel were 123.64 and 86.50 μM, respectively, revealing the higher cellular toxicity and more anti‐skin cancer influences of the nanogel on A431 cells than the conventional gel [143]. On the whole, it seems that nanoformulated lipid carrier gel not only can solve pharmacological limitations of quercetin therapy by elevating aqueous solubility, permeability, managed and sustained release, and drug loading capacity, but also can target skin cancer cells through the reduction of cell viability of cancer cells; however, more in‐depth studies are needed to illustrate the anti‐cancer mechanisms of this nano‐based formulation.

### 6.5 Other Nano‐Based Formulations

In addition to the mentioned nano‐based formulations of quercetin, which have been discussed in skin cancer therapy, there are experimental data recommending some nano‐based drug delivery systems, such as quercetin‐loaded transliposomal gel and optimized mesoporous silica NPs (MSNs) [141, 142].

Liposomes are defined as spherical lipid vesicles, typically ranging from 50 to 500 nm in diameter, that consist of one or more lipid bilayers made by synthetic lipids or emulsifying natural lipids in an aqueous medium [162]. Liposomes are broadly utilized as NPs in nanomedicine owing to several key advantages, including their stability, biocompatibility, simplicity for synthesis, bioavailability, and high drug loading capacity [163, 164, 165]. Besides, the excipients required in these formulations are generally safe [166]. Their unique characteristics, encompassing size and hydrophilic and hydrophobic properties, allow liposomes to effectively encapsulate drug molecules, either within the lipophilic membrane or within the aqueous interior of the vesicles [167]. Ergo, liposomes are known as promising drug delivery systems. Regrettably, liposomes also have some restrictions, like weak targeting, short‐time circulation in vivo, and the likelihood of the fusion leakage of loaded drugs [168, 169].

Lately, Kalam et al. [141] have investigated the dermal usage of fabricated and optimized transliposomes loaded with quercetin (TLQ), based on the ethanol injection method, against melanoma in vitro. This research pointed out that TLQ may solve the pharmacological challenges of quercetin by promoting the skin permeation of quercetin, showing an elevated permeability of quercetin from TLQ and a sustained release pattern. In addition, it was shown that TLQ not only has more antioxidant function but also can decrease the cell viability of B16F10 melanoma cells more effectively than pure quercetin [141].

MSNs are composed of a mesostructured porous network belonging to silicon oxide, which is formed by harnessing the hydrolytic sol‐gel method. This approach comprises the hydrolysis and condensation of silicon alkoxide precursors in basic or acidic circumstances, utilizing a surfactant template [170]. MSNs have suitable biodegradability and biocompatibility and favorable chemical stability, positioning them as a good choice for drug delivery‐related goals [171]. Despite these benefits, MSNs encounter some barriers. For example, the kidney, liver, and other organs may receive encapsulated drugs non‐specifically and confer systemic toxicity [172]. Other limitations of MSNs include compatibility problems, unmanaged degradability, and weak potential for drug encapsulation, which negatively affect their medical uses [173]. Intriguingly, in another scientific effort, optimized MSNs were utilized in order to discover whether this nano‐based platform can boost skin permeation and exert cytotoxic effects on melanoma cells. In this work, MSNs were fabricated via the sol‐gel method and optimized using a Box‐Behnken design. The attained findings revealed that MSNs had favorable sustained profiles in drug release and skin permeation, making them a suitable approach to elevate the effectiveness of quercetin therapy for melanoma. Moreover, the MTT results demonstrated that MSNs loaded with quercetin are more successful in serving as a cytotoxic agent against A375 Melanoma cells [142].

## 7 Clinical Translation: What Are the Challenges?

Although quercetin‐based nanoformulations have recently attracted much attention as potential anti‐cancer therapies in the current era; however, there are some limitations that hinder their clinical applications [174]. The substantial challenges associated with translating quercetin‐based nanoformulations into clinical practice comprise biological problems, safety‐related concerns, biocompatibility, large‐scale manufacturing difficulties, government regulations, intellectual property rights, and unaffordable costs [175]. The transition to the clinical application of quercetin‐based nanoformulations is mainly hindered by biological obstacles and their unclear fate at the diseased site in biological systems [176, 177]. In these systems, nanomaterials interplay with various biomolecules, resulting in the formation of what is known as a “bio‐corona.” The bio‐corona is referred to as different biomolecules, including lipids, peptides, and proteins, that are linked with a NP surface once it is encountered by a biological system. The bio‐corona has a controlling role in the nanodrug efficacy and affects the function of adaptive and natural immunity [178]. Given that NPs and biological aggressors have the same size range, the possibility of interactions with agents related to the immune system increases. So, a main barrier in nanomedicine is the accurate examination of the fate of nano‐based drugs as anti‐cancer agents in the biological system [179]. Overall, certain criteria should be considered to expedite the clinical translation of nanoformulations [1]: The influences of physicochemical features of nanoformulations and their possible mechanisms on their toxicity are still opaque and must be comprehended prior to entering clinical practices, and [2] the benefits provided by nano‐based formulations must outweigh their production costs [180]. So, an achievement in clinical translation of nanoformulations is not always easy to work; it demands in‐depth preclinical research, carefully chosen clinical indications, appropriate design, and faithful implementation of clinical trials.

## 8 Clinical Attempts and Registered Patents

According to data reported from Clinical Trials.gov, the clinical investigation of treatment with quercetin or its nano‐based formulations against skin cancer has not been initiated yet. However, there are some ongoing clinical assessments to show the anti‐cancer potential of quercetin or its combined formulation with dasatinib (a second‐generation inhibitor of tyrosine kinases) against some diseases, including breast cancer, head and neck SCC, and childhood cancer (https://clinicaltrials.gov). Moreover, it has been stated that some clinical trials related to quercetin therapy, either alone or combined, are in the final stage. These clinical studies have investigated the effectiveness of capsulated quercetin, combined quercetin, or a glycosylated form of quercetin (isoquercetin) on some cancers/malignancies, including colorectal cancer, prostate cancer, blood malignancy, non‐small cell lung cancer, and pancreatic cancer (Table 4). These clinical trials are conducted in various countries, mainly in the United States and Iran, and include different phases of clinical research ranging from Phases 1 to 3. The dosages of quercetin or its associated compounds differ from one trial to another. In some research, certain doses, such as 250 mg, 500 mg, or 1000 mg, are prescribed daily for a set period of time, whereas in others, the exact dosage has not been mentioned. In some clinical studies, quercetin is being studied accompanied by other substances like green tea extract, curcumin (a polyphenol obtained from *Curcuma longa*), sulindac (a nonsteroidal anti‐inflammatory drug), and rutin (a quercetin glycoside), showing that these combinations might work together to improve cancer treatment.

**Table 4:** Registered clinical trials (in the completed or terminated status) regarding the effects of quercetin on various cancers/malignancies worldwide (*Source:* https://clinicaltrials.gov).

ID number	Used quercetin	Cancer/Malignancy	Phase	Dose	Country
NCT02195232	Isoquercetin (a glycosylated form of quercetin)	Colorectal cancer, non‐small cell lung cancer, and pancreatic cancer	Phases 2 and 3	500 and 1000 mg/daily for 28 days	United States
NCT01732393	Quercetin capsule	Blood malignancy	Phases 1 and 2	250 mg/daily for 21 days	Iran
NCT01912820	Quercetin and green tea extract	Prostate cancer	Phase 1	—	United States
NCT00003365	Quercetin, curcumin, sulindac, and rutin	Colon cancer	—	—	United States

There is also some patent literature available to aid scientists working on the application of nanoformulations for skin cancer. In Table 5, a collection of registered patents about the performance of nano‐based formulations for skin cancer treatment has been summarized based on data extracted from the Espacenet database (worldwide.Espacenet.com).

**Table 5:** A number of registered patents on nanoformulations for skin cancer treatment (data were obtained from https://worldwide.Espacenet.com).

Type of formulation	Patent number	Intervention	Model	Year	Inventors
Polymeric nanoparticle (DSPE‑PEG)/Photo‑thermal agent	WO2022134862A1	Organic conjugated polymer photo‑thermal agent for the treatment of malignant melanoma	In vitro	2022	Lei et al.
Palladium nanoparticle/Gold nanoparticle/Platinum nanoparticle/Bimetallic gold‑platinum nanoparticle/Bimetallic gold–palladium nanoparticle	US2022218741A1	Synthesis of noble metal oxide nanoparticles by cell‐mediated processes and their biomedical applications	In vitro	2022	Medina et al.
Liposome/Trabectedin, Doxorubicin	WO2022115075A1	Targeted nanoparticles that deliver dual drugs for melanoma therapy	In vitro	2022	Güliz et al.
Liposome/Veirofenib (Vemurafenib), Dacarbazine	CN113244174A	Melanoma chemotherapy drug‐loaded nano‐liposome and its preparation method	In vitro	2021	Qianqian et al.
Polymeric micelle/Acetogenin	ES2826205A1	An approach for constructing a pharmaceutical composition using acetogenins combined with supramolecular polymeric micelles for skin cancer therapy	In vitro	2021	Teresa et al.
Liposome/anti‑PDL1, Catalase	CN110974957A	Utilization of catalase‐entrapped liposomes linked with PD‐L1 antibodies for the development of tumor treatment drugs	In vitro	2020	Shichen et al.
Polymeric nanoparticle (DSPE‑PEG2000)/Temozolomide	CN111481526A	Thermosensitive nanoparticles loaded with a cell‑penetrating peptide‐modified drug and their resistance to melanoma	In vitro	2020	Guan et al.
Carbonyl iron‑sulfur cluster nanoparticle	CN111281858A	Utilization of carbonyl iron‑sulfur clustercompound nanoparticles in drug synthesis	In vivo	2020	Hong et al.
Silver prussian blue nanoparticle	US10231996B2	Silver prussian blue nanoparticles (SPB‑NPs: Ag3[Fe(CN) 6]) coated with a biocampatible polymer	In vivo	2019	Sudip et al.
Gold nanoparticle	US2019142980A1	Cells loaded with gold nanoparticles for the treatment/diagnosis of melanoma	In vivo	2019	Angela et al.
Carbon nanotube	WO2018008825A1	X‑ray brachytherapy system for the treatment of skin cancer and keloid by a carbon nanotube‑based X‑ray tube	—	2018	Oh et al.
Niosome/Doxorubicin	RU2600164C2	A pharmacological gel based on Doxorubicin and organosilicon nanoparticles‑niosomes for skin cancer therapy	—	2016	Aleksandrovich et al.
Organometallic complex (iron‑based)	US2010119608A1	Obtaining pH‑sensitive, acid‑stable metal‑binding nanoparticles	In vivo	2010	Fred et al.

These patents cover a broad range of NPs, comprising liposomes, metallic NPs (e.g., gold, platinum, and palladium), polymeric NPs, and carbon‐based structures like nanotubes. The majority of these formulations are designed to deliver drugs—like temozolomide, doxorubicin, and vemurafenib —in targeted ways to treat melanoma and other skin cancers more effectively. Some patents have noticed the usage of pH‐sensitive organometallic complexes, photothermal agents, and bimetallic NPs, whereas others concentrate on combining NPs with antibodies or peptides in order to target tumor regions better. Collectively, these scientific efforts include lab studies for potential clinical utilization, with specific attention to improving how drugs are delivered, overcoming resistance, and establishing systems that are safe for the human body.

## 9 Barriers to Clinical Translation

No clinical trials specifically evaluating quercetin or its nanoformulations for skin cancer treatment have been conducted to date, despite promising preclinical data. Key barriers include safety concerns, such as quercetin's dose‐dependent pro‐oxidant effects leading to potential organ toxicity (e.g., liver/kidney damage at high doses > 1 g/day) and NP‐related risks like inflammation, oxidative stress, and accumulation in skin or systemic organs.

Regulatory hurdles further complicate translation, as novel nanoformulations require extensive toxicological profiling under FDA/EMA guidelines for nanomaterials, including genotoxicity, biodistribution, and long‐term stability data, which current preclinical studies largely lack. High production costs for scalable, GMP‐compliant nano‐systems (e.g., TiO2 NPs, PLGA‐TPGS) involving complex synthesis, purification, and characterization limit feasibility for large‐scale manufacturing [127].

## 10 Perspectives and Challenges

In the last decades, nanotechnology has been recommended as a new hope to eliminate the limitations of traditional modalities for skin cancer treatment [181]. NPs, thanks to their potential to serve as anti‐cancer agents, tumor‐targeting moieties, skin permeability enhancers, and drug carriers, are generally assumed to be serious candidates to treat this malignancy effectively [182]. As discussed through several experimental studies, nano‐based formulations of quercetin (e.g., titanium dioxide NPs, nanoemulsion, nanostructured lipid carriers, and PLGA‐TPGS NPs) have addressed considerable potential in skin cancer therapy in comparison with conventional strategies. These nanoformulations have changed the landscape of targeted therapy, chemotherapy, and immunotherapy in terms of needed dose, toxicity, therapeutic effectiveness, stability, etc [183]. In particular, for costly therapeutic approaches, like targeted therapy and immunotherapy, enhancing the therapeutic effectiveness with a minimum possible dose is of significance in lowering overall treatment costs [182]. One of the potential therapeutic opportunities for skin cancer is that several other nano‐based formulations have been recommended for skin cancer that can be used for effective and targeted delivery of quercetin to tumor regions [184]. For example, inorganic NPs, including cerium oxide NPs, copper oxide NPs, zinc oxide NPs, platinum, silver NPs, carbon nanotubes, gold NPs, and mesoporous silica, have been offered for skin cancer therapy. These inorganic NPs have several advantages, like small size, biocompatibility, bioactivity, large surface area, and functionalizing capability, which make them appropriate choices for skin cancer treatment [185, 186]. However, they can act as photosensitizing or photothermal agent, which are more commonly used in photodynamic or photothermal therapy [185]. Alongside inorganic NPs, polymer‐based NPs, such as dendrimer, polymersome, polymeric NPs, and polymeric micelle, have also exhibited their ability to adsorb, entrap, conjugate, or load anti‐cancer drugs (lipophilic and hydrophilic drugs, monoclonal antibodies, genes, etc.) for tumor targeting, monitored release, elevated tumor uptake, and safeguard in physiological circumstances [187, 188]. Other suitable types of NPs for skin cancer therapy are lipid‐based NPs. Lipid‐based NPs are unique drug delivery systems that consist of either lipid monolayer (nanostructured lipid carriers and solid lipid NPs) or lipid bilayer (e.g., ethosomes, niosomes, and liposomes) accompanied by aqueous core (ethosomes, niosomes, and liposomes), liquid lipid core (nanostructured lipid carriers), or solid lipid core (solid lipid NPs), wherein drugs can either dissolve or disperse to carry through different administration ways [189, 190]. However, one of the main challenges related to NPs is achieving suitable chemical, physical, pharmacodynamic, and pharmacokinetic features of NPs concerning mass, size, and unique shape. These features are also linked with the secondary features of NPs, like biocompatibility and toxicity [191]. The efficacy of most nanoformulations is affected by some biological challenges, such as biodistribution modulation and controlled NP permeation, across various biological obstacles [192]. Another key challenge for developing nano‐based formulations in the clinic is monitoring the biological fate of NPs. Plus, one of the important barriers of some NPs, especially inorganic NPs, is their prolonged retention in the body, which may increase the possibility of serious side effects, such as chronic toxicity [193].

## 11 Conclusion

This review demonstrates that quercetin exerts potent anti‐cancer effects against skin cancer through multiple mechanisms, including induction of apoptosis, cell cycle arrest, inhibition of proliferation, migration, and invasion, as well as modulation of pathways like IGF‐1, HIF‐1, and RIG‐I in preclinical models of melanoma, SCC, and BCC. Nano‐based formulations, such as TiO2 NPs, nanoemulgels, and PLGA‐TPGS NPs, significantly enhance these effects by improving quercetin's solubility, stability, skin permeability, bioavailability, and targeted delivery, leading to superior cytotoxicity against cancer cells compared to free quercetin while sparing normal cells.

Despite these promising findings, several challenges persist, including quercetin's inherent limitations of poor aqueous solubility, low bioavailability, rapid metabolism, and potential toxicity at high doses. Additionally, nanoformulation issues include particle stability, scalability, cost, and long‐term safety concerns, such as inflammation or organ accumulation. Clinical translation remains hindered by the scarcity of human trials, variability in nanoformulation efficacy across models, and regulatory hurdles for topical or systemic applications.

Future research should prioritize comprehensive preclinical optimization of nano‐quercetin systems, including dose‐response studies, biodistribution analyses, and combination therapies with standard treatments to overcome resistance. Rigorous clinical trials are essential to establish safety, efficacy, optimal dosing, and long‐term outcomes in skin cancer patients, paving the way for regulatory approval and clinical implementation.

## Author Contributions

**Mahtab Khanyabzadeh, Alireza Emamifar, Nikoo Emtiazi, Amir Nazari, Masoumeh Shekarriz, Amin Karami, and Fateme Hashem Beik Mahallati:** conceptualization, methodology, supervision, writing, review and editing. **Rahineh Nomani Lafmejani, Atieh Dariush, Seyed Mohammad Mahdi Rohani, Elaheh Mohseni Vadeghani, and Negin Khoshnood:** resources, writing – review and editing, supervision. **Hamid Reza Ojaghi, Fatemeh Rezaei‐Tazangi, and Reza Arefnezhad:** formal analysis, methodology, visualization, writing – original draft.

## Funding

The authors received no specific funding for this work.

## Disclosure

All authors have read and approved the final version of the manuscript. The corresponding author had full access to all of the data in this study and takes complete responsibility for the integrity of the data and the accuracy of the data analysis.

## Conflicts of Interest

The authors declare no conflicts of interest.

## Transparency Statement

The corresponding author Fatemeh Rezaei‐Tazangi affirms that this manuscript is an honest, accurate, and transparent account of the study being reported; that no important aspects of the study have been omitted; and that any discrepancies from the study as planned (and, if relevant, registered) have been explained.

## Data Availability

The datasets used and analyzed during the current study are available from the authors upon reasonable request.

## References

[1] A. M. Smak Gregoor , T. E. Sangers , L. J. Bakker , et al., “An Artificial Intelligence Based App for Skin Cancer Detection Evaluated in a Population Based Setting,” NPJ Digital Medicine 6, no. 1 (2023): 90. 37210466 10.1038/s41746-023-00831-w PMC10199884

[2] H. M. Balaha and A. E.‐S. Hassan , “Skin Cancer Diagnosis Based on Deep Transfer Learning and Sparrow Search Algorithm,” Neural Computing and Applications 35, no. 1 (2023): 815–853.

[3] M. Mehdizadeh , Z. Mehdizadeh , E. Jafarzadeh , et al., “Skin Cancer Prevention by Nutraceuticals.” Nutraceuticals in Cancer Prevention, Management, and Treatment (Apple Academic Press, 2024), 191–213.

[4] A. K. Gupta , M. Bharadwaj , and R. Mehrotra , “Skin Cancer Concerns in People of Color: Risk Factors and Prevention,” Asian Pacific Journal of Cancer Prevention: APJCP 17, no. 12 (2016): 5257–5264. 28125871 10.22034/APJCP.2016.17.12.5257 PMC5454668

[5] N. H. Matthews , K. Fitch , W.‐Q. Li , et al., “Exposure to Trace Elements and Risk of Skin Cancer: A Systematic Review of Epidemiologic Studies,” Cancer Epidemiology, Biomarkers & Prevention: A Publication of the American Association for Cancer Research, cosponsored by the American Society of Preventive Oncology 28, no. 1 (2019): 3–21. 10.1158/1055-9965.EPI-18-0286 PMC6324965 30297516

[6] X. Geng , X. Qiu , J. Gao , et al., “CREB1 Regulates KPNA2 by Inhibiting mir‐495‐3p Transcription to Control Melanoma Progression: The Role of the CREB1/miR‐495‐3p/KPNA2 Axis in Melanoma Progression,” BMC Molecular and Cell Biology 23, no. 1 (2022): 57. 36522613 10.1186/s12860-022-00446-1 PMC9756468

[7] F. Rezaei‐Tazangi , A. Forutan Mirhosseini , A. Fathi , H. Roghani‐Shahraki , R. Arefnezhad , and F. Vasei , “Herbal and Nano‐Based Herbal Medicine: New Insights Into Their Therapeutic Aspects Against Periodontitis,” Avicenna Journal of Phytomedicine 14, no. 4 (2024): 430–454. 38952769 10.22038/AJP.2023.23261 PMC11179182

[8] A. Rasul , F. M. Millimouno , W. Ali Eltayb , M. Ali , J. Li , and X. Li , “Pinocembrin: A Novel Natural Compound With Versatile Pharmacological and Biological Activities,” BioMed Research International 2013 (2013): 1–9. 10.1155/2013/379850 PMC3747598 23984355

[9] S. W. Aziz and M. H. Aziz , “Protective Molecular Mechanisms of Resveratrol in UVR‐Induced Skin Carcinogenesis,” Photodermatology, Photoimmunology & Photomedicine 34, no. 1 (2018): 35–41. 10.1111/phpp.12336 28767162

[10] D. Lelli , C. Pedone , and A. Sahebkar , “Curcumin and Treatment of Melanoma: The Potential Role of MicroRNAs,” Biomedicine & Pharmacotherapy = Biomedecine & Pharmacotherapie 88 (2017): 832–834. 28167449 10.1016/j.biopha.2017.01.078

[11] F. Soll , C. Ternent , I. M. Berry , D. Kumari , and T. C. Moore , “Quercetin Inhibits Proliferation and Induces Apoptosis of B16 Melanoma Cells In Vitro,” ASSAY and Drug Development Technologies 18, no. 6 (2020): 261–268. 32799543 10.1089/adt.2020.993

[12] J. Milutinov , V. Krstonošić , D. Ćirin , M. Hadnađev , M. Đanić , and N. Pavlović , “Development and Evaluation of Quercetin Topical Emulgels: Physicochemical and Rheological Properties, Stability and Sun Protective Potential,” Journal of Molecular Liquids 417 (2025): 126568.

[13] S. Kowalski , J. Karska , M. Tota , K. Skinderowicz , J. Kulbacka , and M. Drąg‐Zalesińska , “Natural Compounds in Non‐Melanoma Skin Cancer: Prevention and Treatment,” Molecules 29, no. 3 (2024): 728. 38338469 10.3390/molecules29030728 PMC10856721

[14] F. Rezaee‐Tazangi , N. Varaa , L. Khorsandi , and M. Abbaspour , “Effects of Silymarin‐Loaded Polylactic‐Co‐Glycolic Acid Nanoparticles on Osteoarthritis in Rats,” Iranian Journal of Science and Technology, Transactions A: Science 44, no. 3 (2020): 605–614.

[15] E. Siljamäki , P. Riihilä , U. Suwal , et al., “Inhibition of TGF‐β Signaling, Invasion, and Growth of Cutaneous Squamous Cell Carcinoma by PLX8394,” Oncogene 42 (2023): 3633–3647. 37864034 10.1038/s41388-023-02863-8 PMC10691969

[16] A. G. Marzuka and S. E. Book , “Basal Cell Carcinoma: Pathogenesis, Epidemiology, Clinical Features, Diagnosis, Histopathology, and Management,” The Yale Journal of Biology and Medicine 88, no. 2 (2015): 167–179. 26029015 PMC4445438

[17] M. I. Achatz , M. C. G. Coloma , and E. de Albuquerque Cavalcanti Callegaro , “Risk Factors for Skin Cancer.” Oncodermatology: An Evidence‐Based, Multidisciplinary Approach to Best Practices (Springer, 2023), 37–55.

[18] M. Zambrano‐Román , J. R. Padilla‐Gutiérrez , Y. Valle , J. F. Muñoz‐Valle , and E. Valdés‐Alvarado , “Non‐Melanoma Skin Cancer: A Genetic Update and Future Perspectives,” Cancers 14, no. 10 (2022): 2371. 35625975 10.3390/cancers14102371 PMC9139429

[19] D. Didona , G. Paolino , U. Bottoni , and C. Cantisani , “Non Melanoma Skin Cancer Pathogenesis Overview,” Biomedicines 6, no. 1 (2018): 6. 29301290 10.3390/biomedicines6010006 PMC5874663

[20] A. Nazari , P. Osati , S. Seifollahy Fakhr , et al., “New Emerging Therapeutic Strategies Based on Manipulation of the Redox Regulation Against Therapy Resistance in Cancer,” Antioxidants & Redox Signaling (2024): 1–39. 10.1089/ars.2023.0491 39506926

[21] C. Zilberg , J. G. Lyons , R. Gupta , and D. L. Damian , “The Immune Microenvironment in Basal Cell Carcinoma,” Annals of Dermatology 35, no. 4 (2023): 243–255. 37550225 10.5021/ad.22.042 PMC10407341

[22] J.‐W. Tjiu , J.‐S. Chen , C.‐T. Shun , et al., “Tumor‐Associated Macrophage‐Induced Invasion and Angiogenesis of Human Basal Cell Carcinoma Cells by Cyclooxygenase‐2 Induction,” Journal of Investigative Dermatology 129, no. 4 (2009): 1016–1025. 18843292 10.1038/jid.2008.310

[23] H. J. Lee , Y. J. Hong , and M. Kim , “Angiogenesis in Chronic Inflammatory Skin Disorders,” International Journal of Molecular Sciences 22, no. 21 (2021): 12035. 34769465 10.3390/ijms222112035 PMC8584589

[24] M. Azimi , M. S. Manavi , M. Afshinpour , et al., “Emerging Immunologic Approaches as Cancer Anti‐Angiogenic Therapies,” Clinical and Translational Oncology 27 (2024): 1–20. 39294514 10.1007/s12094-024-03667-2

[25] A. T. Westin , L. G. Gardinassi , E. G. Soares , J. S. Da Silva , E. A. Donadi , and C. Da Silva Souza , “HLA‐G, Cytokines, and Cytokine Receptors in the Non‐Aggressive Basal Cell Carcinoma Microenvironment,” Archives of Dermatological Research 314 (2022): 247–256. 33811555 10.1007/s00403-021-02218-x

[26] I. Elamin , R. D. Zecević , D. Vojvodić , L. Medenica , and M. D. Pavlović , “Cytokine Concentrations in Basal Cell Carcinomas of Different Histological Types and Localization,” Acta Dermatovenerologica Alpina, Pannonica et Adriatica 17, no. 2 (2008): 55–59. 18709290

[27] B. S. Maner , L. Dupuis , A. Su , et al., “Overview of Genetic Signaling Pathway Interactions Within Cutaneous Malignancies,” Journal of Cancer Metastasis and Treatment 6: N/A–N/A (2020).

[28] Z. Apalla , D. Nashan , R. B. Weller , and X. Castellsagué , “Skin Cancer: Epidemiology, Disease Burden, Pathophysiology, Diagnosis, and Therapeutic Approaches,” Dermatology and Therapy 7 (2017): 5–19. 28150105 10.1007/s13555-016-0165-y PMC5289116

[29] M. Piipponen , P. Riihilä , L. Nissinen , and V. M. Kähäri , “The Role of p53 in Progression of Cutaneous Squamous Cell Carcinoma,” Cancers 13, no. 18 (2021): 4507. 34572732 10.3390/cancers13184507 PMC8466956

[30] G. Vallini , L. Calabrese , C. Canino , et al., “Signaling Pathways and Therapeutic Strategies in Advanced Basal Cell Carcinoma,” Cells 12, no. 21 (2023): 2534. 37947611 10.3390/cells12212534 PMC10647618

[31] I. A. Bednarski , M. Ciążyńska , K. Wódz , et al., “Hippo Signaling Pathway as a New Potential Target in Non‐Melanoma Skin Cancers: A Narrative Review,” Life 11, no. 7 (2021): 680. 34357052 10.3390/life11070680 PMC8306788

[32] M. Tampa , S. R. Georgescu , M. I. Mitran , et al., “Current Perspectives on the Role of Matrix Metalloproteinases in the Pathogenesis of Basal Cell Carcinoma,” Biomolecules 11, no. 6 (2021): 903. 34204372 10.3390/biom11060903 PMC8235174

[33] L. Dall'Olmo , N. Papa , N. C. Surdo , I. Marigo , and S. Mocellin , “Alpha‐Melanocyte Stimulating Hormone (α‐MSH): Biology, Clinical Relevance and Implication in Melanoma,” Journal of Translational Medicine 21, no. 1 (2023): 562. 37608347 10.1186/s12967-023-04405-y PMC10463388

[34] M. Jarrar and A. El‐Shafey , “The Paradox of Ectopic Melanin Synthesis in Adipose: Potential Mechanism, Benefits 828 and Perspectives in Abating Obesity Complications,” Journal of Obesity & Weight Loss Therapy 8, no. 363 (2018): 2.

[35] S. Strashilov and A. Yordanov , “Aetiology and Pathogenesis of Cutaneous Melanoma: Current Concepts and Advances,” International Journal of Molecular Sciences 22, no. 12 (2021): 6395. 34203771 10.3390/ijms22126395 PMC8232613

[36] F. L. Meyskens , P. J. Farmer , and H. Anton‐Culver , “Etiologic Pathogenesis of Melanoma,” Clinical Cancer Research 10, no. 8 (2004): 2581–2583. 15102657 10.1158/1078-0432.ccr-03-0638

[37] M. Ottaviano , E. Giunta , M. Tortora , et al., “BRAF Gene and Melanoma: Back to the Future,” International Journal of Molecular Sciences 22, no. 7 (2021): 3474. 33801689 10.3390/ijms22073474 PMC8037827

[38] M. Mandalà , B. Merelli , and D. Massi , “Nras in Melanoma: Targeting the Undruggable Target,” Critical Reviews in Oncology/Hematology 92, no. 2 (2014): 107–122. 24985059 10.1016/j.critrevonc.2014.05.005

[39] J. F. Abdo , A. Sharma , and R. Sharma , “Role of Heredity in Melanoma Susceptibility: A Primer for the Practicing Surgeon,” Surgical Clinics of North America 100, no. 1 (2020): 13–28. 31753108 10.1016/j.suc.2019.09.006

[40] G. C. Leonardi , L. Falzone , R. Salemi , et al., “Cutaneous Melanoma: From Pathogenesis to Therapy,” International Journal of Oncology 52, no. 4 (2018): 1071–1080. 29532857 10.3892/ijo.2018.4287 PMC5843392

[41] K. R. Lee , J. S. Lee , Y. R. Kim , I. G. Song , and E. K. Hong , “Polysaccharide From Inonotus Obliquus Inhibits Migration and Invasion in B16‐F10 Cells by Suppressing MMP‑2 and MMP‑9 via Downregulation of NF‐κB Signaling Pathway,” Oncology Reports 31, no. 5 (2014): 2447–2453. 24677090 10.3892/or.2014.3103

[42] L. Falzone , R. Salemi , S. Travali , et al., “MMP‐9 Overexpression Is Associated With Intragenic Hypermethylation of MMP9 Gene in Melanoma,” Aging 8, no. 5 (2016): 933–944. 27115178 10.18632/aging.100951 PMC4931845

[43] E.‐M. Tomou , P. Papakyriakopoulou , E.‐M. Saitani , G. Valsami , N. Pippa , and H. Skaltsa , “Recent Advances in Nanoformulations for Quercetin Delivery,” Pharmaceutics 15, no. 6 (2023): 1656. 37376104 10.3390/pharmaceutics15061656 PMC10302355

[44] E. Cecerska‐Heryć , Z. Wiśniewska , N. Serwin , et al., “Can Compounds of Natural Origin Be Important in Chemoprevention? Anticancer Properties of Quercetin, Resveratrol, and Curcumin—A Comprehensive Review,” International Journal of Molecular Sciences 25, no. 8 (2024): 4505. 38674092 10.3390/ijms25084505 PMC11050349

[45] E.‐M. Tomou , P. Papakyriakopoulou , H. Skaltsa , G. Valsami , and N. P. E. Kadoglou , “Bio‐Actives From Natural Products With Potential Cardioprotective Properties: Isolation, Identification, and Pharmacological Actions of Apigenin, Quercetin, and Silibinin,” Molecules 28, no. 5 (2023): 2387. 36903630 10.3390/molecules28052387 PMC10005323

[46] S. R. Kumar and N. Ponpandian , “Quercetin Bioflavonoids Derived From Phytomedicinal Compounds for Targeted Drug Delivery and Their Antioxidant Properties.” Nanophytomedicine (CRC Press, 2022), 221–234.

[47] E. J. Carrillo‐Martinez , F. Y. Flores‐Hernández , A. M. Salazar‐Montes , H. F. Nario‐Chaidez , and L. D. Hernández‐Ortega , “Quercetin, a Flavonoid With Great Pharmacological Capacity,” Molecules 29, no. 5 (2024): 1000. 38474512 10.3390/molecules29051000 PMC10935205

[48] X. Cai , Z. Fang , J. Dou , A. Yu , and G. Zhai , “Bioavailability of Quercetin: Problems and Promises,” Current Medicinal Chemistry 20, no. 20 (2013): 2572–2582. 23514412 10.2174/09298673113209990120

[49] N. Suganthy , K. P. Devi , S. F. Nabavi , N. Braidy , and S. M. Nabavi , “Bioactive Effects of Quercetin in the Central Nervous System: Focusing on the Mechanisms of Actions,” Biomedicine & Pharmacotherapy = Biomedecine & Pharmacotherapie 84 (2016): 892–908. 27756054 10.1016/j.biopha.2016.10.011

[50] Y. Hai , Y. Zhang , Y. Liang , et al., “Advance on the Absorption, Metabolism, and Efficacy Exertion of Quercetin and Its Important Derivatives: Absorption, Metabolism and Function of Quercetin,” Food Frontiers 1, no. 4 (2020): 420–434.

[51] K. Kandemir , M. Tomas , D. J. McClements , and E. Capanoglu , “Recent Advances on the Improvement of Quercetin Bioavailability,” Trends in Food Science & Technology 119 (2022): 192–200.

[52] X. Chen , O. Q. P. Yin , Z. Zuo , and M. S. S. Chow , “Pharmacokinetics and Modeling of Quercetin and Metabolites,” Pharmaceutical Research 22 (2005): 892–901. 15948033 10.1007/s11095-005-4584-1

[53] C. Bologa Foods That Promote Health. Foods That Harm, Foods That Promote Health: A Biochemical and Nutritional Perspective in Health and Disease Prevention Irvine, CA, USA: Universal Publishers Inc. 2021:181–213.

[54] I.‐L. Chen , Y.‐J. Tsai , C.‐M. Huang , and T.‐H. Tsai , “Lymphatic Absorption of Quercetin and Rutin in Rat and Their Pharmacokinetics in Systemic Plasma,” Journal of Agricultural and Food Chemistry 58, no. 1 (2010): 546–551. 19916501 10.1021/jf9026124

[55] R. Arefnezhad , S. S. Amin , A. Mohammdi , et al., “Quercetin and Its Nanoformulations as Promising Agents for Lung Cancer Treatment: A Focus on Molecular Mechanisms,” Journal of Drug Delivery Science and Technology 99 (2024): 105933.

[56] D. Sangeetha , “Therapeutic Potentials and Targeting Strategies of Quercetin on Cancer Cells: Challenges and Future Prospects,” Phytomedicine 133 (2024): 155902. 39059266 10.1016/j.phymed.2024.155902

[57] G. B. Lee , Y. Kim , K. E. Lee , R. Vinayagam , M. Singh , and S. G. Kang , “Anti‐Inflammatory Effects of Quercetin, Rutin, and Troxerutin Result From the Inhibition of NO Production and the Reduction of COX‐2 Levels in RAW 264.7 Cells Treated With LPS,” Applied Biochemistry and Biotechnology 196 (2024): 8431–8452. 39096472 10.1007/s12010-024-05003-4

[58] S. Roy , P. Ezati , A. Khan , and J.‐W. Rhim , “New Opportunities and Advances in Quercetin‐Added Functional Packaging Films for Sustainable Packaging Applications: A Mini‐Review,” Critical Reviews in Food Science and Nutrition 64, no. 23 (2024): 8464–8479. 37074182 10.1080/10408398.2023.2200553

[59] F. Samadi , M. S. Kahrizi , F. Heydari , et al., “Quercetin and Osteoarthritis: A Mechanistic Review on the Present Documents,” Pharmacology 107, no. 9–10 (2022): 464–471. 35793647 10.1159/000525494

[60] Y. Almuhanna , A. Alshalani , H. AlSudais , et al., “Antibacterial, Antibiofilm, and Wound Healing Activities of Rutin and Quercetin and Their Interaction With Gentamicin on Excision Wounds in Diabetic Mice,” Biology 13, no. 9 (2024): 676. 39336103 10.3390/biology13090676 PMC11429020

[61] M. Ali , M. Hassan , S. A. Ansari , H. M. Alkahtani , L. S. Al‐Rasheed , and S. A. Ansari , “Quercetin and Kaempferol as Multi‐Targeting Antidiabetic Agents Against Mouse Model of Chemically Induced Type 2 Diabetes,” Pharmaceuticals 17, no. 6 (2024): 757. 38931424 10.3390/ph17060757 PMC11206732

[62] M. A. Fawzy , G. Nasr , F. E. M. Ali , and M. Fathy , “Quercetin Potentiates the Hepatoprotective Effect of Sildenafil and/or Pentoxifylline Against Intrahepatic Cholestasis: Role of Nrf2/ARE, TLR4/NF‐κB, and NLRP3/IL‐1β Signaling Pathways,” Life Sciences 314 (2023): 121343. 36592787 10.1016/j.lfs.2022.121343

[63] D. A. Espírito‐Santo , G. S. Cordeiro , L. S. Santos , et al., “Cardioprotective Effect of the Quercetin on Cardiovascular Remodeling and Atherosclerosis in Rodents Fed a High‐Fat Diet: A Systematic Review,” Chemico‐Biological Interactions 384 (2023): 110700. 37690744 10.1016/j.cbi.2023.110700

[64] M.‐C. Chiang , T.‐Y. Tsai , and C.‐J. Wang , “The Potential Benefits of Quercetin for Brain Health: A Review of Anti‐Inflammatory and Neuroprotective Mechanisms,” International Journal of Molecular Sciences 24, no. 7 (2023): 6328. 37047299 10.3390/ijms24076328 PMC10094159

[65] Y.‐F. Zeng , J.‐Y. Li , X.‐Y. Wei , et al., “Preclinical Evidence of Reno‐Protective Effect of Quercetin on Acute Kidney Injury: A Meta‐Analysis of Animal Studies,” Frontiers in Pharmacology 14 (2023): 1310023. 38186644 10.3389/fphar.2023.1310023 PMC10770850

[66] N. Yadav , A. Tripathi , A. Parveen , S. Parveen , and M. Banerjee , “PLGA‐Quercetin Nano‐Formulation Inhibits Cancer Progression via Mitochondrial Dependent Caspase‐3, 7 and Independent FoxO1 Activation With Concomitant PI3K/AKT Suppression,” Pharmaceutics 14, no. 7 (2022): 1326. 35890222 10.3390/pharmaceutics14071326 PMC9323198

[67] I. Deepika and P. K. Maurya , “Health Benefits of Quercetin in Age‐Related Diseases,” Molecules 27, no. 8 (2022): 2498. 35458696 10.3390/molecules27082498 PMC9032170

[68] X. Zang , M. Cheng , X. Zhang , and X. Chen , “Quercetin Nanoformulations: A Promising Strategy for Tumor Therapy,” Food & Function 12, no. 15 (2021): 6664–6681. 34152346 10.1039/d1fo00851j

[69] A. Chitkara , B. Mangla , P. Kumar , S. Javed , W. Ahsan , and H. Popli , “Design‐Of‐Experiments (DoE)‐Assisted Fabrication of Quercetin‐Loaded Nanoemulgel and Its Evaluation Against Human Skin Cancer Cell Lines,” Pharmaceutics 14, no. 11 (2022): 2517. 36432708 10.3390/pharmaceutics14112517 PMC9692577

[70] S. Andres , S. Pevny , R. Ziegenhagen , et al., “Safety Aspects of the Use of Quercetin as a Dietary Supplement,” Molecular Nutrition & Food Research 62, no. 1 (2018): 1700447. 10.1002/mnfr.201700447 29127724

[71] P. Singh , S. Sharma , and S. K. Rath , “A Versatile Flavonoid Quercetin: Study of Its Toxicity and Differential Gene Expression in the Liver of Mice,” Phytomedicine Plus 2, no. 1 (2022): 100148.

[72] N. T. Lu , C. M. Crespi , N. M. Liu , et al., “A Phase I Dose Escalation Study Demonstrates Quercetin Safety and Explores Potential for Bioflavonoid Antivirals in Patients With Chronic Hepatitis C,” Phytotherapy Research 30, no. 1 (2016): 160–168. 26621580 10.1002/ptr.5518 PMC5590840

[73] D. A. Shoskes , S. I. Zeitlin , A. Shahed , and J. Rajfer , “Quercetin in Men With Category III Chronic Prostatitis: A Preliminary Prospective, Double‐Blind, Placebo‐Controlled Trial,” Urology 54, no. 6 (1999): 960–963. 10604689 10.1016/s0090-4295(99)00358-1

[74] J. K. Dunnick and J. R. Halley , “Toxicity and Carcinogenicity Studies of Quercetin, a Natural Component of Foods,” Toxicological Sciences 19, no. 3 (1992): 423–431. 10.1016/0272-0590(92)90181-g 1459373

[75] N. Ito , A. Hagiwara , S. Tamano , et al., “Lack of Carcinogenicity of Quercetin in F344/DuCrj Rats,” Japanese Journal of Cancer Research 80, no. 4 (1989): 317–325. 2501248 10.1111/j.1349-7006.1989.tb02313.x PMC5917733

[76] N. T. Program , “Toxicology and Carcinogenesis Studies of Quercetin (CAS No. 117‐39‐5) in F344 Rats (Feed Studies),” National Toxicology Program Technical Report Series 409 (1992): 1–171. 12621521

[77] J.‐S. Choi , Y.‐J. Piao , and K. W. Kang , “Effects of Quercetin on the Bioavailability of Doxorubicin in Rats: Role of CYP3A4 and P‐Gp Inhibition by Quercetin,” Archives of Pharmacal Research 34 (2011): 607–613. 21544726 10.1007/s12272-011-0411-x

[78] V. R. Challa , P. Ravindra Babu , S. R. Challa , B. Johnson , and C. Maheswari , “Pharmacokinetic Interaction Study Between Quercetin and Valsartan in Rats and In Vitro Models,” Drug Development and Industrial Pharmacy 39, no. 6 (2013): 865–872. 22670860 10.3109/03639045.2012.693502

[79] J.‐S. Choi , B.‐W. Jo , and Y.‐C. Kim , “Enhanced Paclitaxel Bioavailability After Oral Administration of Paclitaxel or Prodrug to Rats Pretreated With Quercetin,” European Journal of Pharmaceutics and Biopharmaceutics 57, no. 2 (2004): 313–318. 15018990 10.1016/j.ejpb.2003.11.002

[80] S. Shin , J. Choi , and X. Li , “Enhanced Bioavailability of Tamoxifen After Oral Administration of Tamoxifen With Quercetin in Rats,” International Journal of Pharmaceutics 313, no. 1–2 (2006): 144–149. 16516418 10.1016/j.ijpharm.2006.01.028

[81] Y.‐H. Wang , P.‐D. L. Chao , S.‐L. Hsiu , K.‐C. Wen , and Y.‐C. Hou , “Lethal Quercetin‐Digoxin Interaction in Pigs,” Life Sciences 74, no. 10 (2004): 1191–1197. 14697403 10.1016/j.lfs.2003.06.044

[82] P. R. Babu , K. N. Babu , P. L. H. Peter , K. Rajesh , and P. J. Babu , “Influence of Quercetin on the Pharmacokinetics of Ranolazine in Rats and In Vitro Models,” Drug Development and Industrial Pharmacy 39, no. 6 (2013): 873–879. 22817837 10.3109/03639045.2012.707209

[83] T. Bansal , A. Awasthi , M. Jaggi , R. K. Khar , and S. Talegaonkar , “Pre‐Clinical Evidence for Altered Absorption and Biliary Excretion of Irinotecan (CPT‐11) in Combination With Quercetin: Possible Contribution of P‐Glycoprotein,” Life Sciences 83, no. 7–8 (2008): 250–259. 18619980 10.1016/j.lfs.2008.06.007

[84] M. A. Nguyen , P. Staubach , S. Wolffram , and P. Langguth , “Effect of Single‐Dose and Short‐Term Administration of Quercetin on the Pharmacokinetics of Talinolol in Humans–Implications for the Evaluation of Transporter‐Mediated Flavonoid–Drug Interactions,” European Journal of Pharmaceutical Sciences 61 (2014): 54–60. 24472704 10.1016/j.ejps.2014.01.003

[85] M. A. Nguyen , P. Staubach , S. Wolffram , and P. Langguth , “The Influence of Single‐Dose and Short‐Term Administration of Quercetin on the Pharmacokinetics of Midazolam in Humans,” Journal of Pharmaceutical Sciences 104, no. 9 (2015): 3199–3207. 25988261 10.1002/jps.24500

[86] K.‐A. Kim , P.‐W. Park , and J.‐Y. Park , “Short‐Term Effect of Quercetin on the Pharmacokinetics of Fexofenadine, a Substrate of P‐Glycoprotein, in Healthy Volunteers,” European Journal of Clinical Pharmacology 65 (2009): 609–614. 19221726 10.1007/s00228-009-0627-6

[87] L. X. Wu , C. X. Guo , W. Q. Chen , et al., “Inhibition of the Organic Anion‐Transporting Polypeptide 1B1 by Quercetin: An in Vitro and In Vivo Assessment,” British Journal of Clinical Pharmacology 73, no. 5 (2012): 750–757. 22114872 10.1111/j.1365-2125.2011.04150.x PMC3403202

[88] A. Singh , P. S. Naidu , and S. K. Kulkarni , “Quercetin Potentiates L‐Dopa Reversal of Drug‐Induced Catalepsy in Rats: Possible COMT/MAO Inhibition,” Pharmacology 68, no. 2 (2003): 81–88. 12711835 10.1159/000069533

[89] K. J. P. Rocha‐Brito , S. P. Clerici , H. G. Cordeiro , et al., “Quercetin Increases Mitochondrial Proteins (VDAC and SDH) and Downmodulates AXL and PIM‐1 Tyrosine Kinase Receptors in NRAS Melanoma Cells,” Biological Chemistry 403, no. 3 (2022): 293–303. 34854272 10.1515/hsz-2021-0261

[90] H. R. Rezvani , N. Ali , L. J. Nissen , et al., “HIF‐1α in Epidermis: Oxygen Sensing, Cutaneous Angiogenesis, Cancer, and Non‐Cancer Disorders,” Journal of Investigative Dermatology 131, no. 9 (2011): 1793–1805. 21633368 10.1038/jid.2011.141

[91] A. Huma and D. Savita , “Quercetin Attenuates the Development of 7, 12‐Dimethyl Benz (A) Anthracene (DMBA) and Croton Oil‐Induced Skin Cancer in Mice,” Journal of Biomedical Research 29, no. 2 (2015): 139. 25859269 10.7555/JBR.29.20130025 PMC4389114

[92] N. H. Aljarba , H. Ali , and S. Alkahtani , “Synergistic Dose Permutation of Isolated Alkaloid and Sterol for Anticancer Effect on Young Swiss Albino Mice,” Drug Design, Development and Therapy 15 (2021): 4043–4052. 34588765 10.2147/DDDT.S322769 PMC8476085

[93] M. Jung , S. Y. Bu , K.‐H. Tak , J.‐E. Park , and E. Kim , “Anticarcinogenic Effect of Quercetin by Inhibition of Insulin‐Like Growth Factor (IGF)−1 Signaling in Mouse Skin Cancer,” Nutrition Research and Practice 7, no. 6 (2013): 439–445. 24353828 10.4162/nrp.2013.7.6.439 PMC3865265

[94] Y. Gao , C. Li , T. Xue , et al., “Quercetin Mediated TET1 Expression Through MicroRNA‐17 Induced Cell Apoptosis in Melanoma Cells,” Biochemical Genetics 61, no. 2 (2023): 762–777. 36136257 10.1007/s10528-022-10286-5

[95] Y.‐F. He , B.‐Z. Li , Z. Li , et al., “Tet‐Mediated Formation of 5‐Carboxylcytosine and Its Excision by TDG in Mammalian DNA,” Science 333, no. 6047 (2011): 1303–1307. 21817016 10.1126/science.1210944 PMC3462231

[96] Y. Yu , J. Qi , J. Xiong , et al., “Epigenetic Co‐Deregulation of EZH2/TET1 Is a Senescence‐Countering, Actionable Vulnerability in Triple‐Negative Breast Cancer,” Theranostics 9, no. 3 (2019): 761–777. 30809307 10.7150/thno.29520 PMC6376470

[97] D. Peng , L. Chen , Y. Sun , et al., “Melanoma Suppression by Quercein Is Correlated With Rig‐I and Type I Interferon Signaling,” Biomedicine & Pharmacotherapy = Biomedecine & Pharmacotherapie 125 (2020): 109984. 32066042 10.1016/j.biopha.2020.109984

[98] H. Hundsberger , A. Stierschneider , V. Sarne , et al., “Concentration‐Dependent Pro‐And Antitumor Activities of Quercetin in Human Melanoma Spheroids: Comparative Analysis of 2D and 3D Cell Culture Models,” Molecules 26, no. 3 (2021): 717. 33573155 10.3390/molecules26030717 PMC7866537

[99] S.‐H. Kim , E.‐S. Yoo , J.‐S. Woo , et al., “Antitumor and Apoptotic Effects of Quercetin on Human Melanoma Cells Involving JNK/P38 MAPK Signaling Activation,” European Journal of Pharmacology 860 (2019): 172568. 31348906 10.1016/j.ejphar.2019.172568

[100] A. Sturza , I. Pavel , S. Ancușa , et al., “Quercetin Exerts an Inhibitory Effect on Cellular Bioenergetics of the B164A5 Murine Melanoma Cell Line,” Molecular and Cellular Biochemistry 447 (2018): 103–109. 29380243 10.1007/s11010-018-3296-x

[101] K. A. Turner , J. M. Manouchehri , and M. Kalafatis , “Sensitization of Recombinant Human Tumor Necrosis Factor‐Related Apoptosis‐Inducing Ligand‐Resistant Malignant Melanomas by Quercetin,” Melanoma Research 28, no. 4 (2018): 277–285. 29596115 10.1097/CMR.0000000000000447 PMC6039425

[102] R. A. Rafiq , A. Quadri , L. A. Nazir , K. Peerzada , B. A. Ganai , and S. A. Tasduq , “A Potent Inhibitor of Phosphoinositide 3‐Kinase (PI3K) and Mitogen Activated Protein (MAP) Kinase Signalling, Quercetin (3, 3’, 4’, 5, 7‐Pentahydroxyflavone) Promotes Cell Death in Ultraviolet (UV)‐B‐Irradiated B16F10 Melanoma Cells,” PLoS One 10, no. 7 (2015): e0131253. 26148186 10.1371/journal.pone.0131253 PMC4493061

[103] H.‐H. Cao , C.‐Y. Cheng , T. Su , et al., “Quercetin Inhibits HGF/C‐Met Signaling and HGF‐Stimulated Melanoma Cell Migration and Invasion,” Molecular Cancer 14 (2015): 103. 25971889 10.1186/s12943-015-0367-4 PMC4435529

[104] H.‐H. Cao , A. K.‐W. Tse , H.‐Y. Kwan , et al., “Quercetin Exerts Anti‐Melanoma Activities and Inhibits STAT3 Signaling,” Biochemical Pharmacology 87, no. 3 (2014): 424–434. 24275163 10.1016/j.bcp.2013.11.008

[105] T. Thangasamy , S. Sittadjody , G. C. Mitchell , et al., “Quercetin Abrogates Chemoresistance in Melanoma Cells by Modulating ΔNp73,” BMC Cancer 10, no. 1 (2010): 282. 20540768 10.1186/1471-2407-10-282 PMC2895613

[106] T. Thangasamy , S. Sittadjody , S. Lanza‐Jacoby , P. R. Wachsberger , K. H. Limesand , and R. Burd , “Quercetin Selectively Inhibits Bioreduction and Enhances Apoptosis in Melanoma Cells That Overexpress Tyrosinase,” Nutrition and Cancer 59, no. 2 (2007): 258–268. 18001220 10.1080/01635580701499545

[107] K. Horváthová , I. Chalupa , L. Šebová , D. Tóthová , and A. Vachálková , “Protective Effect of Quercetin and Luteolin in Human Melanoma HMB‐2 Cells,” Mutation Research/Genetic Toxicology and Environmental Mutagenesis 565, no. 2 (2005): 105–112. 10.1016/j.mrgentox.2004.08.013 15661608

[108] H. Nagata , S. Takekoshi , R. Takeyama , T. Homma , and R. Yoshiyuki Osamura , “Quercetin Enhances Melanogenesis by Increasing the Activity and Synthesis of Tyrosinase in Human Melanoma Cells and in Normal Human Melanocytes,” Pigment Cell Research 17, no. 1 (2004): 66–73. 14717847 10.1046/j.1600-0749.2003.00113.x

[109] X.‐M. Zhang , S.‐P. Huang , and Q. Xu , “Quercetin Inhibits the Invasion of Murine Melanoma B16‐BL6 Cells by Decreasing pro‐MMP‐9 via the PKC Pathway,” Cancer Chemotherapy and Pharmacology 53 (2004): 82–88. 14593496 10.1007/s00280-003-0702-0

[110] X. Zhang , Q. Xu , and I. Saiki , “Quercetin Inhibits the Invasion and Mobility of Murine Melanoma B16‐BL6 Cells Through Inducing Apoptosis via Decreasing Bcl‐2 Expression,” Clinical & Experimental Metastasis 18 (2000): 415–421. 11467774 10.1023/a:1010960615370

[111] A. J. Vargas and R. Burd , “Hormesis and Synergy: Pathways and Mechanisms of Quercetin in Cancer Prevention and Management,” Nutrition Reviews 68, no. 7 (2010): 418–428. 20591109 10.1111/j.1753-4887.2010.00301.x

[112] S. Shanavas , A. Priyadharsan , S. Karthikeyan , et al., “Green Synthesis of Titanium Dioxide Nanoparticles Using Phyllanthus Niruri Leaf Extract and Study on Its Structural, Optical and Morphological Properties,” Materials Today: Proceedings 26 (2020): 3531–3534.

[113] C. Lin , B. D. Choudhury , R. Ybarra , et al., “Tailoring the Structure–Property Relationships of Innovative Flowerlike TiO2 Structures in a Fiber‐Shaped Dye‐Sensitized Solar Cell,” ACS Applied Energy Materials 7, no. 6 (2024): 2329–2337.

[114] K. G. Pavithra , P. S. Kumar , V. Jaikumar , and P. S. Jaikumar , “Removal of Colorants From Wastewater: A Review on Sources and Treatment Strategies,” Journal of Industrial and Engineering Chemistry 75 (2019): 1–19.

[115] C. Li and M. Tang , “The Toxicological Effects of Nano Titanium Dioxide on Target Organs and Mechanisms of Toxicity,” Journal of Applied Toxicology 44, no. 2 (2024): 152–164. 37655586 10.1002/jat.4534

[116] Y. Wang , M. Xu , J. Li , and T. Zhang , “Photocatalytic Degradation of Organic Pollutants Using Al/TiO2 Composites Under Visible Light,” Environmental Engineering Science 41, no. 5 (2024): 204–215.

[117] M. U. Younas , A. Usman , A. R. Kashif , A. Zahoor , and F. Haider , “Green Synthesis of TiO2 NPs Using Agave Americana Leaves: Antioxidant, Cytotoxicity, and Photocatalytic Activity,” ChemistrySelect 9, no. 44 (2024): e202404050.

[118] R. Chandoliya , S. Sharma , V. Sharma , R. Joshi , and I. Sivanesan , “Titanium Dioxide Nanoparticle: A Comprehensive Review on Synthesis, Applications and Toxicity,” Plants 13, no. 21 (2024): 2964. 39519883 10.3390/plants13212964 PMC11547906

[119] Y. Birinci , J. H. Niazi , O. Aktay‐Çetin , and H. Basaga , “Quercetin in the Form of a Nano‐Antioxidant (QTiO2) Provides Stabilization of Quercetin and Maximizes Its Antioxidant Capacity in the Mouse Fibroblast Model,” Enzyme and Microbial Technology 138 (2020): 109559. 32527528 10.1016/j.enzmictec.2020.109559

[120] F. Eker , H. Duman , E. Akdaşçi , et al., “A Comprehensive Review of Nanoparticles: From Classification to Application and Toxicity,” Molecules 29, no. 15 (2024): 3482. 39124888 10.3390/molecules29153482 PMC11314082

[121] S. Mallakpour , C. M. Hussain , D. Thomas , et al., “Consumer Nanoproducts for Biomedical Applications,” Handbook of Consumer Nanoproducts 6 (2021): 1–27.

[122] M. Marzi , M. Osanloo , M. K. Vakil , et al., “Applications of Metallic Nanoparticles in the Skin Cancer Treatment,” BioMed Research International 2022, no. 1 (2022): 2346941. 36420097 10.1155/2022/2346941 PMC9678447

[123] T. Ponraj , R. Vivek , M. Paulpandi , et al., “Mitochondrial Dysfunction‐Induced Apoptosis in Breast Carcinoma Cells Through a pH‐Dependent Intracellular Quercetin NDDS of PVPylated‐TiO 2 NPs,” Journal of Materials Chemistry B 6, no. 21 (2018): 3555–3570. 32254451 10.1039/c8tb00769a

[124] F. M. P. Tonelli , F. C. P. Tonelli , and H. G. Cordeiro , “TiO2 Nanoparticles in Cancer Therapy as Nanocarriers in Paclitaxel's Delivery and Nanosensitizers in Phototherapies and/or Sonodynamic Therapy,” Current Pharmaceutical Biotechnology 25, no. 2 (2023): 133–143. 10.2174/1389201024666230518124829 37202892

[125] A. Chahardoli , F. Jalilian , Y. Shokoohinia , and A. Fattahi , “The Role of Quercetin in the Formation of Titanium Dioxide Nanoparticles for Nanomedical Applications,” Toxicology In Vitro 87 (2023): 105538. 36535556 10.1016/j.tiv.2022.105538

[126] Y. Li , J. Yang , and X. Sun , “Reactive Oxygen Species‐Based Nanomaterials for Cancer Therapy,” Frontiers in Chemistry 9 (2021): 650587. 33968899 10.3389/fchem.2021.650587 PMC8100441

[127] X.‐R. Li , L. Qi , X.‐W. Zhang , C. Wei , B. Yu , and T.‐L. Pei , “Quercetin and Nano‐Derivatives: Potential and Challenges in Cancer Therapy,” International Journal of Nanomedicine 20 (2025): 6701–6720. 40444010 10.2147/IJN.S509877 PMC12120254

[128] K. Sghier , M. Mur , F. Veiga , A. C. Paiva‐Santos , and P. C. Pires , “Novel Therapeutic Hybrid Systems Using Hydrogels and Nanotechnology: A Focus on Nanoemulgels for the Treatment of Skin Diseases,” Gels 10, no. 1 (2024): 45. 38247768 10.3390/gels10010045 PMC10815052

[129] E. Sánchez‐López , M. Guerra , J. Dias‐Ferreira , et al., “Current Applications of Nanoemulsions in Cancer Therapeutics,” Nanomaterials 9, no. 6 (2019): 821. 31159219 10.3390/nano9060821 PMC6632105

[130] P. Sengupta and B. Chatterjee , “Potential and Future Scope of Nanoemulgel Formulation for Topical Delivery of Lipophilic Drugs,” International Journal of Pharmaceutics 526, no. 1–2 (2017): 353–365. 28461261 10.1016/j.ijpharm.2017.04.068

[131] G. Aggarwal , B. Dhawan , and S. Harikumar , “Enhanced Transdermal Permeability of Piroxicam Through Novel Nanoemulgel Formulation,” International Journal of Pharmaceutical Investigation 4, no. 2 (2014): 65. 25006551 10.4103/2230-973X.133053 PMC4083536

[132] M. S. Algahtani , M. Z. Ahmad , and J. Ahmad , “Nanoemulgel for Improved Topical Delivery of Retinyl Palmitate: Formulation Design and Stability Evaluation,” Nanomaterials 10, no. 5 (2020): 848. 32353979 10.3390/nano10050848 PMC7711631

[133] Y. Yin , B. Hu , X. Yuan , L. Cai , H. Gao , and Q. Yang , “Nanogel: A Versatile Nano‐Delivery System for Biomedical Applications,” Pharmaceutics 12, no. 3 (2020): 290. 32210184 10.3390/pharmaceutics12030290 PMC7151186

[134] H. Choudhury , B. Gorain , M. Pandey , et al., “Recent Update on Nanoemulgel as Topical Drug Delivery System,” Journal of Pharmaceutical Sciences 106, no. 7 (2017): 1736–1751. 28412398 10.1016/j.xphs.2017.03.042

[135] N. Sharma , M. Bansal , S. Visht , P. Sharma , and G. Kulkarni , “Nanoemulsion: A New Concept of Delivery System,” Chronicles of Young Scientists 1, no. 2 (2010): 2–6.

[136] S. Bhavesh and C. Shah , “Nanoemulgel: A Comprehensive Review on the Recent Advances in Topical Drug Delivery,” Pharma Science Monitor 7, no. 2 (2016): 346–355.

[137] J. Gutiérrez , C. González , A. Maestro , I. Solè , C. Pey , and J. N., “Nano‐Emulsions: New Applications and Optimization of Their Preparation,” Current Opinion in Colloid & Interface Science 13, no. 4 (2008): 245–251.

[138] G. C. Aithal , U. Y. Nayak , C. Mehta , et al., “Localized In Situ Nanoemulgel Drug Delivery System of Quercetin for Periodontitis: Development and Computational Simulations,” Molecules 23, no. 6 (2018): 1363. 29882751 10.3390/molecules23061363 PMC6099597

[139] R. Moral and E. Escrich , “Influence of Olive Oil and Its Components on Breast Cancer: Molecular Mechanisms,” Molecules 27, no. 2 (2022): 477. 35056792 10.3390/molecules27020477 PMC8780060

[140] C. L. Dora , L. F. Costa Silva , L. Mazzarino , et al., “Oral Delivery of a High Quercetin Payload Nanosized Emulsion: In Vitro and In Vivo Activity Against B16‐F10 Melanoma,” Journal of Nanoscience and Nanotechnology 16, no. 2 (2016): 1275–1281. 27433577 10.1166/jnn.2016.11675

[141] M. A. Kalam , R. Ali , A. Alhowyan , A. Ahmad , M. Iqbal , and M. Raish , “Quercetin‐Loaded Transliposomal Gel for Effective Management of Skin Cancer: In Vitro and Cell Line Efficacy Studies,” Journal of Drug Delivery Science and Technology 96 (2024): 105659.

[142] P. Zahedi , P. Ebrahimnejad , M. Seyedabadi , and A. Babaei , “Optimized Mesoporous Silica Nanoparticles for Delivery of Curcumin and Quercetin: Enhanced Skin Permeation and Cytotoxicity Against A375 Melanoma Cells,” Journal of Cluster Science 36, no. 2 (2025): 50.

[143] M. Imran , M. K. Iqubal , K. Imtiyaz , et al., “Topical Nanostructured Lipid Carrier Gel of Quercetin and Resveratrol: Formulation, Optimization, In Vitro and Ex Vivo Study for the Treatment of Skin Cancer,” International Journal of Pharmaceutics 587 (2020): 119705. 32738456 10.1016/j.ijpharm.2020.119705

[144] A. Bagde , K. Patel , A. Mondal , et al., “Combination of UVB Absorbing Titanium Dioxide and Quercetin Nanogel for Skin Cancer Chemoprevention,” AAPS PharmSciTech 20 (2019): 240. 31250221 10.1208/s12249-019-1424-x

[145] A. Singh , S. Parikh , N. Sethi , S. Patel , N. Modi , and K. Patel , “Nanoparticle Formulations: A Sustainable Approach to Biodegradable and Non‐Biodegradable Products,” Nanocarrier Vaccines: Biopharmaceutics‐Based Fast Track Development 419 (2024): 95–151.

[146] S. I. Thamake , S. L. Raut , Z. Gryczynski , A. P. Ranjan , and J. K. Vishwanatha , “Alendronate Coated Poly‐Lactic‐Co‐Glycolic Acid (PLGA) Nanoparticles for Active Targeting of Metastatic Breast Cancer,” Biomaterials 33, no. 29 (2012): 7164–7173. 22795543 10.1016/j.biomaterials.2012.06.026

[147] X. Zeng , W. Tao , L. Mei , L. Huang , C. Tan , and S.‐S. Feng , “Cholic Acid‐Functionalized Nanoparticles of Star‐Shaped PLGA‐Vitamin E TPGS Copolymer for Docetaxel Delivery to Cervical Cancer,” Biomaterials 34, no. 25 (2013): 6058–6067. 23694904 10.1016/j.biomaterials.2013.04.052

[148] T. Peng , Y. Huang , X. Feng , et al., “TPGS/Hyaluronic Acid Dual‐Functionalized PLGA Nanoparticles Delivered Through Dissolving Microneedles for Markedly Improved Chemo‐Photothermal Combined Therapy of Superficial Tumor,” Acta Pharmaceutica Sinica B 11, no. 10 (2021): 3297–3309. 34729317 10.1016/j.apsb.2020.11.013 PMC8546669

[149] R. Sun , Y. Chen , Y. Pei , et al., “The Drug Release of PLGA‐Based Nanoparticles and Their Application in Treatment of Gastrointestinal Cancers,” Heliyon 10, no. 18 (2024): e38165. 39364250 10.1016/j.heliyon.2024.e38165 PMC11447355

[150] L. Mu and S. S. Feng , “A Novel Controlled Release Formulation for the Anticancer Drug Paclitaxel (Taxol®): PLGA Nanoparticles Containing Vitamin E TPGS,” Journal of Controlled Release 86, no. 1 (2003): 33–48. 12490371 10.1016/s0168-3659(02)00320-6

[151] M. Wang and M. Thanou , “Targeting Nanoparticles to Cancer,” Pharmacological Research 62, no. 2 (2010): 90–99. 20380880 10.1016/j.phrs.2010.03.005

[152] H. Zhu , H. Chen , X. Zeng , et al., “Co‐Delivery of Chemotherapeutic Drugs With Vitamin E TPGS by Porous PLGA Nanoparticles for Enhanced Chemotherapy Against Multi‐Drug Resistance,” Biomaterials 35, no. 7 (2014): 2391–2400. 24360574 10.1016/j.biomaterials.2013.11.086

[153] X. Zhu , X. Zeng , X. Zhang , et al., “The Effects of Quercetin‐Loaded PLGA‐TPGS Nanoparticles on Ultraviolet B‐Induced Skin Damages In Vivo,” Nanomedicine: Nanotechnology, Biology and Medicine 12, no. 3 (2016): 623–632. 26656634 10.1016/j.nano.2015.10.016

[154] M. Elmowafy and M. M. Al‐Sanea , “Nanostructured Lipid Carriers (NLCs) as Drug Delivery Platform: Advances in Formulation and Delivery Strategies,” Saudi Pharmaceutical Journal 29, no. 9 (2021): 999–1012. 34588846 10.1016/j.jsps.2021.07.015 PMC8463508

[155] M. A. Iqbal , S. Md , J. K. Sahni , S. Baboota , S. Dang , and J. Ali , “Nanostructured Lipid Carriers System: Recent Advances in Drug Delivery,” Journal of Drug Targeting 20, no. 10 (2012): 813–830. 22931500 10.3109/1061186X.2012.716845

[156] P. Jaiswal , B. Gidwani , and A. Vyas , “Nanostructured Lipid Carriers and Their Current Application in Targeted Drug Delivery,” Artificial Cells, Nanomedicine, and Biotechnology 44, no. 1 (2016): 27–40. 24813223 10.3109/21691401.2014.909822

[157] S. Sadegh Malvajerd , A. Azadi , Z. Izadi , et al., “Brain Delivery of Curcumin Using Solid Lipid Nanoparticles and Nanostructured Lipid Carriers: Preparation, Optimization, and Pharmacokinetic Evaluation,” ACS Chemical Neuroscience 10, no. 1 (2018): 728–739. 30335941 10.1021/acschemneuro.8b00510

[158] E. B. Souto , A. J. Almeida , and R. H. Müller , “Lipid Nanoparticles (SLN®, NLC®) for Cutaneous Drug Delivery:Structure, Protection and Skin Effects,” Journal of Biomedical Nanotechnology 3, no. 4 (2007): 317–331.

[159] B. Iqbal , J. Ali , and S. Baboota , “Silymarin Loaded Nanostructured Lipid Carrier: From Design and Dermatokinetic Study to Mechanistic Analysis of Epidermal Drug Deposition Enhancement,” Journal of Molecular Liquids 255 (2018): 513–529.

[160] S. Saleem , M. K. Iqubal , S. Garg , J. Ali , and S. Baboota , “Trends in Nanotechnology‐Based Delivery Systems for Dermal Targeting of Drugs: An Enticing Approach to Offset Psoriasis,” Expert Opinion on Drug Delivery 17, no. 6 (2020): 817–838. 32315216 10.1080/17425247.2020.1758665

[161] K. Sghier , M. Mur , F. Veiga , A. C. Paiva‐Santos , and P. C. Pires , “Novel Therapeutic Hybrid Systems Using Hydrogels and Nanotechnology: A Focus on Nanoemulgels for the Treatment of Skin Diseases,” Gels 10, no. 1 (2024): 45. 38247768 10.3390/gels10010045 PMC10815052

[162] H. Nsairat , D. Khater , U. Sayed , F. Odeh , A. Al Bawab , and W. Alshaer , “Liposomes: Structure, Composition, Types, and Clinical Applications,” Heliyon 8, no. 5 (2022): e09394. 35600452 10.1016/j.heliyon.2022.e09394 PMC9118483

[163] G. T. Noble , J. F. Stefanick , J. D. Ashley , T. Kiziltepe , and B. Bilgicer , “Ligand‐Targeted Liposome Design: Challenges and Fundamental Considerations,” Trends in Biotechnology 32, no. 1 (2014): 32–45. 24210498 10.1016/j.tibtech.2013.09.007

[164] A. Hafner , J. Lovrić , G. P. Lakoš , and I. Pepić , “Nanotherapeutics in the EU: An Overview on Current State and Future Directions,” International Journal of Nanomedicine 19 (2014): 1005–1023. 24600222 10.2147/IJN.S55359 PMC3933707

[165] D. J. McClements and J. Rao , “Food‐Grade Nanoemulsions: Formulation, Fabrication, Properties, Performance, Biological Fate, and Potential Toxicity,” Critical Reviews in Food Science and Nutrition 51, no. 4 (2011): 285–330. 21432697 10.1080/10408398.2011.559558

[166] T. Chen , T. Gong , T. Zhao , Y. Fu , Z. Zhang , and T. Gong , “A Comparison Study Between Lycobetaine‐Loaded Nanoemulsion and Liposome Using nRGD as Therapeutic Adjuvant for Lung Cancer Therapy,” European Journal of Pharmaceutical Sciences 111 (2018): 293–302. 28966099 10.1016/j.ejps.2017.09.041

[167] S. Fathi and A. K. Oyelere , “Liposomal Drug Delivery Systems for Targeted Cancer Therapy: Is Active Targeting the Best Choice?,” Future Medicinal Chemistry 8, no. 17 (2016): 2091–2112. 27774793 10.4155/fmc-2016-0135

[168] A. Salvaggio , A. Privitera , G. Iannello , and M. V. Brundo , “Innovations in Skin Aging: Biomimetic Exosomes Versus Natural Exosomes,” bioRxiv 330 (2025): 631233.

[169] J.‐S. Kim , “Liposomal Drug Delivery System,” Journal of Pharmaceutical Investigation 46 (2016): 387–392. 10.1007/s40005-016-0258-8 PMC7100357 32226640

[170] M. Mladenović , S. Jarić , M. Mundžić , A. Pavlović , I. Bobrinetskiy , and N. Knežević , “Biosensors for Cancer Biomarkers Based on Mesoporous Silica Nanoparticles,” Biosensors 14, no. 7 (2024): 326. 39056602 10.3390/bios14070326 PMC11274377

[171] W. Weisany , S. Yousefi , S. P. Soufiani , D. Pashang , D. J. McClements , and M. Ghasemlou , “Mesoporous Silica Nanoparticles: A Versatile Platform for Encapsulation and Delivery of Essential Oils for Food Applications,” Advances in Colloid and Interface Science 325 (2024): 103116. 38430728 10.1016/j.cis.2024.103116

[172] X. Sha , Y. Dai , X. Song , S. Liu , S. Zhang , and J. Li , “The Opportunities and Challenges of Silica Nanomaterial for Atherosclerosis,” International Journal of Nanomedicine 16 (2021): 701–714. 33536755 10.2147/IJN.S290537 PMC7850448

[173] A. Nair , R. Chandrashekhar H., C. M. Day , et al., “Polymeric Functionalization of Mesoporous Silica Nanoparticles: Biomedical Insights,” International Journal of Pharmaceutics 660 (2024): 124314. 38862066 10.1016/j.ijpharm.2024.124314

[174] T. Sharma , D. Singh , A. Mahapatra , P. Mohapatra , S. Sahoo , and S. K. Sahoo , “Advancements in Clinical Translation of Flavonoid Nanoparticles for Cancer Treatment,” OpenNano 8 (2022): 100074.

[175] S. Hua , M. B. C. De Matos , J. M. Metselaar , and G. Storm , “Current Trends and Challenges in the Clinical Translation of Nanoparticulate Nanomedicines: Pathways for Translational Development and Commercialization,” Frontiers in Pharmacology 9 (2018): 790. 30065653 10.3389/fphar.2018.00790 PMC6056679

[176] T. Lammers , F. Kiessling , W. E. Hennink , and G. Storm , “Drug Targeting to Tumors: Principles, Pitfalls and (Pre‐) Clinical Progress,” Journal of Controlled Release 161, no. 2 (2012): 175–187. 21945285 10.1016/j.jconrel.2011.09.063

[177] M. Neagu , Z. Piperigkou , K. Karamanou , et al., “Protein Bio‐Corona: Critical Issue in Immune Nanotoxicology,” Archives of Toxicology 91 (2017): 1031–1048. 27438349 10.1007/s00204-016-1797-5 PMC5316397

[178] A. K. Simon , G. A. Hollander , and A. McMichael , “Evolution of the Immune System in Humans From Infancy to Old Age,” Proceedings of the Royal Society B: Biological Sciences 282, no. 1821 (2015): 20143085. 10.1098/rspb.2014.3085 PMC4707740 26702035

[179] A. E.‐M. El‐Kenawy , C. Constantin , S. M. Hassan , et al., “Nanomedicine in Melanoma: Current Trends and Future Perspectives,” Exon Publications 30 (2017): 143–159. 29461779

[180] H. Liu , P. van Nooten , L. Deng , and W. Cui , “Clinical Translation of Nanomaterials,” Theranostic Bionanomaterials 122 (2019): 75–111.

[181] V. M. Prajapat , S. Mahajan , P. G. Paul , et al., “Nanomedicine: A Pragmatic Approach for Tackling Melanoma Skin Cancer,” Journal of Drug Delivery Science and Technology 83 (2023): 104394.

[182] L. Zeng , B. H. J. Gowda , M. G. Ahmed , et al., “Advancements in Nanoparticle‐Based Treatment Approaches for Skin Cancer Therapy,” Molecular Cancer 22, no. 1 (2023): 10. 36635761 10.1186/s12943-022-01708-4 PMC9835394

[183] P. R. Kumbhar , P. Kumar , A. Lasure , R. Velayutham , and D. Mandal , “An Updated Landscape on Nanotechnology‐Based Drug Delivery, Immunotherapy, Vaccinations, Imaging, and Biomarker Detections for Cancers: Recent Trends and Future Directions With Clinical Success,” Discover Nano 18, no. 1 (2023): 156. 38112935 10.1186/s11671-023-03913-6 PMC10730792

[184] M. K. Iqubal , M. A. Khan , N. B. Agarwal , J. Ali , and S. Baboota , “Nanoformulations‐Based Advancement in the Delivery of Phytopharmaceuticals for Skin Cancer Management,” Journal of Drug Delivery Science and Technology 66 (2021): 102912.

[185] S. Muthukrishnan , A. V. Anand , K. Palanisamy , G. Gunasangkaran , A. K. Ravi , and B. Balasubramanian , “Novel Organic and Inorganic Nanoparticles as a Targeted Drug Delivery Vehicle in Cancer Treatment,” Emerging Nanomaterials for Advanced Technologies 22 (2022): 117–161.

[186] E. Carazo , A. Borrego‐Sánchez , F. García‐Villén , et al., “Advanced Inorganic Nanosystems for Skin Drug Delivery,” The Chemical Record 18, no. 7–8 (2018): 891–899. 29314634 10.1002/tcr.201700061

[187] H. Choudhury , B. Gorain , M. Pandey , R. K. Khurana , and P. Kesharwani , “Strategizing Biodegradable Polymeric Nanoparticles to Cross the Biological Barriers for Cancer Targeting,” International Journal of Pharmaceutics 565 (2019): 509–522. 31102804 10.1016/j.ijpharm.2019.05.042

[188] Z. Zhang , P. C. Tsai , T. Ramezanli , and B. B. Michniak‐Kohn , “Polymeric Nanoparticles‐Based Topical Delivery Systems for the Treatment of Dermatological Diseases,” WIREs Nanomedicine and Nanobiotechnology 5, no. 3 (2013): 205–218. 23386536 10.1002/wnan.1211 PMC3631287

[189] A. Aziz , U. Rehman , A. Sheikh , M. A. S. Abourehab , and P. Kesharwani , “Lipid‐Based Nanocarrier Mediated CRISPR/Cas9 Delivery for Cancer Therapy,” Journal of Biomaterials Science, Polymer Edition 34, no. 3 (2023): 398–418. 36083788 10.1080/09205063.2022.2121592

[190] N. Dhiman , R. Awasthi , B. Sharma , H. Kharkwal , and G. T. Kulkarni , “Lipid Nanoparticles as Carriers for Bioactive Delivery,” Frontiers in Chemistry 9 (2021): 580118. 33981670 10.3389/fchem.2021.580118 PMC8107723

[191] A. Wicki , D. Witzigmann , V. Balasubramanian , and J. Huwyler , “Nanomedicine in Cancer Therapy: Challenges, Opportunities, and Clinical Applications,” Journal of Controlled Release 200 (2015): 138–157. 25545217 10.1016/j.jconrel.2014.12.030

[192] A. C. Anselmo and S. Mitragotri , “Nanoparticles in the Clinic,” Bioengineering & Translational Medicine 1 (2016): 10–29. 29313004 10.1002/btm2.10003 PMC5689513

[193] M. Arif , A. F. Nawaz , S. Ullah khan , et al., “Nanotechnology‐Based Radiation Therapy to Cure Cancer and the Challenges in Its Clinical Applications,” Heliyon 9, no. 6 (2023): e17252. 37389057 10.1016/j.heliyon.2023.e17252 PMC10300336

